# Isolation and Characterization of Bioactive Compounds from *Saccharomonospora* sp. CMS18 and Their Antifungal Properties

**DOI:** 10.3390/md22120539

**Published:** 2024-11-30

**Authors:** Soohyun Um, Hyeongju Jeong, Ji-Eun Park, Jeongwon Seo, Sang Heon Jung, Munhyung Bae, Kyung-Tae Lee, Kyuho Moon

**Affiliations:** 1 College of Pharmacy, Yonsei University, Incheon 21983, Republic of Korea; soohyunum@yonsei.ac.kr; 2 Research Institute of Pharmaceutical Sciences, College of Pharmacy, Chonnam National University, Gwangju 61186, Republic of Korea; 217843@jnu.ac.kr (H.J.); jeongwon.0522@gmail.com (J.S.); 3 Korea Research Institute for Veterinary Biologics, Iksan 54531, Republic of Korea; doraemong16@naver.com; 4 Korea Zoonosis Research Institute, Jeonbuk National University, Iksan 54531, Republic of Korea; 5 College of Pharmacy, Gachon University, Incheon 21936, Republic of Korea; fly6778@gachon.ac.kr (S.H.J.); baemoon89@gachon.ac.kr (M.B.); 6 College of Pharmacy, Kyung Hee University, Seoul 02447, Republic of Korea; 7 Department of Biomedical and Pharmaceutical Sciences, Graduate School, Kyung Hee University, Seoul 02447, Republic of Korea

**Keywords:** tidal mudflat, structural determination, secondary metabolite, antifungal agent

## Abstract

In this study, metagenomic analysis was employed to investigate the bacterial communities in the Muan tidal mudflat of the Republic of Korea. We used metagenomic analysis to identify the microbial community in tidal soil dominated by Proteobacteria. From this environment, the bacterial strain, *Saccharomonospora* sp. CMS18, was isolated and yielded two previously unknown compounds, penipaline D (**3**) and *N*-acetyl-dimethylallyltryptophan (**4**). The chemical structures of the isolated compounds along with 6-dimethylallyl-indole (**1**), 6-dimethylallyltryptophan (**2**), penipaline D (**3**), and *N*-acetyl-dimethylallyltryptophan (**4**) were structurally investigated using HR-ESI-MS and NMR spectroscopy. The isolated compound 6-dimethylallyl-indole (**1**) demonstrated broad-spectrum antifungal activity, with IC_50_ value of 0.04 mM against *Candida glabrata* and 0.35 mM against both *Candida albicans* and *Cryptococcus neoformans*. Additionally, it exhibited additive interaction with caspofungin against *C. albicans*.

## 1. Introduction

The important roles played by tidal mudflats in the decomposition of organic matter and the cycling of nutrients have set them apart as dynamic coastal ecosystems [1]. These environments are home to a wide variety of microorganisms that actively participate in the decomposition of organic materials, thereby facilitating the recycling of vital nutrients [2,3]. Although the ecological roles of tidal flats have historically been the focus of research, they are also acknowledged as significant stores of bioactive natural products, particularly secondary metabolites produced by local microorganisms [4]. These substances have shown a great deal of promise for use in pharmaceutical applications; the development of new antibiotics and anticancer treatments is one example of this [5].

Actinomycetes are microorganisms that thrive in tidal mudflats and are distinguished by their abundant production of a variety of secondary metabolites [6,7]. For example, the well-known actinomycete *Streptomyces* is a source of several common antibiotics, including erythromycin, tetracycline, and streptomycin [8,9,10]. *Streptomyces* species are responsible for the synthesis of nystatin, an antifungal agent, and doxorubicin, an important antitumor agent [11,12]. Similarly, *Saccharomonospora* species also produce diverse bioactive compounds, many of which have been developed as therapeutic agents [13,14]. However, little is known about the secondary metabolites produced by actinomycetes in tidal mudflat environments, especially in special places such as Korea’s Muan tidal mudflat, which is regarded as one of the world’s largest tidal flats [15].

Recent developments in metagenomic methodologies have provided a more profound understanding of the microbial diversity in these environments [16]. For instance, metagenomic sequencing has enabled a comprehensive definition of microbial communities inhabiting tidal flats and the identification of microbes that may be beneficial for the discovery of natural products [17,18]. In this study, the bioactive potential of actinomycetes, particularly those found in the Muan tidal mudflat, was investigated using a combination of traditional methods of natural product separation and metagenomic analysis.

Although the ecological importance of tidal mudflats has been widely acknowledged, research on the bioactive potential of these microbial communities is lacking. To address the gap in research on microbial bioactive compounds from tidal mudflats, we isolated *Saccharomonospora* sp. CMS18 from the Muan tidal mudflat. Three novel compounds were identified using advanced analytical techniques such as nuclear magnetic resonance (NMR) spectroscopy and high-resolution electrospray ionization mass spectrometry (HR-ESI-MS). Additionally, the biological activities of these compounds were assessed to highlight their potential therapeutic applications by assessing their antifungal efficacy against major invasive fungal pathogens.

## 2. Results and Discussion

### 2.1. Composition Analysis of Metagenomics Sequencing

**Figure 1:**
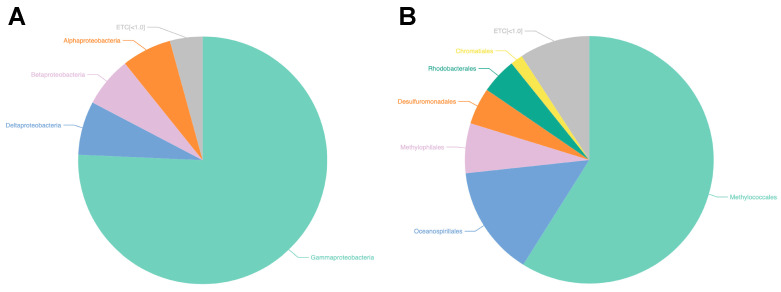
Metagenomics targeted bacteria taxon of the mud sample. The data show Microbiome Taxonomic Profile (MTP) for the mud sample at the level of (**A**) class and (**B**) order.

By utilizing the genomic DNA isolated from the sediment, 424.8 bp read lengths (with a minimum of 148 and a maximum of 449) of the V3-V4 hypervariable sections of the bacterial 16S rRNA gene were amplified. Using the Illumina MiSeq platform, 55,298 amplicons were sequenced, and 41,765 quality-filtered reads were produced (low-quality amplicons = 3562, non-target amplicons = 78, and chimeric amplicons, 9893). The number of reads that could be classified as belonging to a specific species was 37,280 (89.3%), and 673 bacterial species were identified. Good’s coverage, which reflects the database coverage rate of a sample, was calculated as the ratio of OTUs, excluding singletons, in the entire read. For the sediment, the values of alpha diversity, also known as species diversity, which exists in a certain place and in a specific sample, were 1, 631.8, 1520.5 for Chao1 and 1727. The sediment had a Shannon index value of 2.903, with a margin of error of 0.026. This value represented the number of species and the effect of evenness. The phylogenetic diversity of the mud sample was found to be 1920 after calculating the shortest distances between the nodes of the system diagram. The discovered OTUs were categorized into the following groups: 32 phyla (41,765 OTUs), 74 classes, 142 orders, 249 families, 551 genera, and 783 species. Proteobacteria, the dominant taxon, were identified in 96.6% of the total 32 phyla, including Bacteroidetes (1.1%), Acidobacteria (0.34%), Chloroflexi (0.3%), and Actinobacteria (0.2%). Gammaproteobacteria was the most prevalent phylum within the phylum Proteobacteria, accounting for 75.7% of all readings. This was followed by Deltaproteobacteria and Betaproteobacteria, which accounted for 7.0% and 6.6% of all reads, respectively, at the class level. At the order level, Methylococcales comprised 58.9% of the population. This was followed by the Methylophilales (6.5%) and Oceanospirillales (14.4%) (Figure 1).

Metagenomic analysis indicated that Actinobacteria, which encompasses genera such as *Streptomyces* and *Saccharomonospora*, constituted only 0.2% of the overall bacterial community in the sediment. The low proportion of Actinobacteria may be attributed to limitations of metagenomic sequencing, which often fails to capture slow-growing or low-abundance microorganisms in environmental samples. Additionally, Actinobacteria may require particular growth conditions, and their metabolic activity may be inhibited under specific environmental conditions, rendering them less detectable via metagenomic techniques. Despite the low abundance of Actinobacteria observed in the metagenomic analysis, the isolation of *Saccharomonospora* sp. CMS18, using traditional culturing techniques, highlights the importance of integrating both cultivation-dependent and cultivation-independent methods. This discrepancy underscores the limitations of metagenomics in identifying bioactive microorganisms and emphasizes the need for culturing techniques to fully assess the metabolic potential of tidal flat microbial communities.

### 2.2. Structural Elucidation

**Figure 2:**
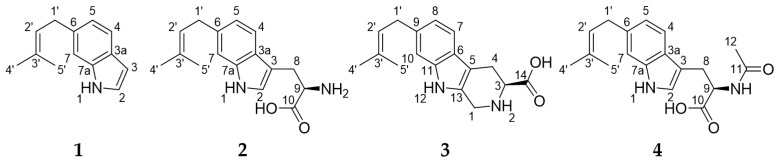
Structures of 6-dimetyhlallyl-indole (**1**), 6-dimethylallyltryptophan (**2**), penipaline D (**3**), and *N*-Acetyl-6-dimethylallyltryptophan (**4**).

6-Dimethylallyl-indole (**1**) and 6-dimethylallyltryptophan (**2**) were isolated from actinomycete *Saccharomonospora* sp. CMS18 and its chemical structure were determined by liquid LC-MS, high-resolution electrospray ionization mass spectrometry (HR-ESI-MS), and nuclear magnetic resonance (NMR) analysis. Compounds **1** and **2** were obtained as colorless and yellow oils, respectively, and their molecular formulae were determined to be C_13_H_15_N and C_16_H_20_N_2_O_2_ by HR-ESI-MS (*m*/*z* 186.1277 [M + H]^+^ calculated for C_13_H_16_N *m*/*z* 186.1277 [M + H]^+^ and *m*/*z* 273.1604 [M + H]^+^ calculated for C_16_H_21_N_2_O_2_, *m*/*z* 273.1597 [M + H]^+^) together with ^1^H and ^13^C NMR data (Table 1 and Table 2). The chemical structures of **1** and **2** were confirmed using 1D (^1^H and ^13^C) and 2D (COSY, HSQC, and HMBC) NMR spectroscopy, with the spectra recorded in methanol-*d*_4_ (CD_3_OD-*d*_4_). The chemical structures of **1** and **2** were identified as 6-dimethylallyl-indole and 6-dimethylallyltryptophan by comparing the NMR data with the literature (Figure 2) [19,20,21].

**Figure 3:**
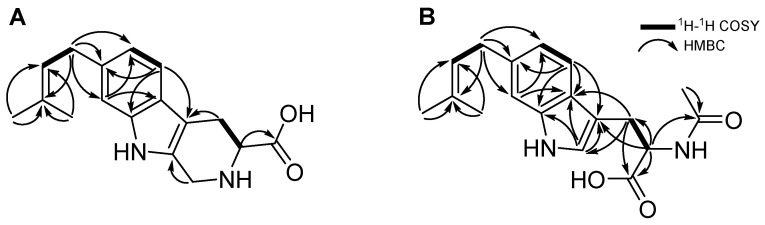
Key ^1^H-^1^H COSY and HMBC correlations of (**A**) **3** and (**B**) **4**.

Penipaline D (**3**) was isolated as a yellow oil, and its molecular formula was determined to be C_17_H_20_N_2_O_2_ based on HR-ESI-MS (*m*/*z* 285.1588 [M + H]^+^, calculated for C_17_H_21_N_2_O_2_, *m*/*z* 285.1597 [M + H]^+^) in combination with ^1^H and ^13^C NMR spectroscopic data (Table 3). A comparison of the NMR data of **3** with those of **2** and **4** indicated that the structures of the compounds were very similar in the dimethylallyl group and indole moiety, except for the cyclized tryptophan in **3**. The HMBC correlations from H_2_-4 (δ_H_ 3.41 and 3.03) to C-5 (δ_C_ 107.7); from H_2_-1 (δ_H_ 4.39) to C-13 (δ_C_ 126.1); and from H-3 (δ_H_ 3.94) to C-14 (δ_C_ 173.9) indicated **3** has cyclomethyltryptophan instead of the tryptophan (Figure 2 and Figure 3). Thus, the structure of **3** was determined as a β-carboline derivative, named penipaline D (**3**) [22].

*N*-Acetyl-6-dimethylallyl-l-tryptophan (**4**) was obtained as a colorless oil, and its molecular formula was assigned as C_18_H_22_N_2_O_3_ by HR-ESI-MS (*m*/*z* 315.1713 [M + H]^+^, calculated for C_18_H_23_N_2_O_3_, *m*/*z* 315.1703 [M + H]^+^) in combination with ^1^H and ^13^C NMR data (Table 4). The chemical structure of **4** was elucidated using the 1D and 2D NMR spectra acquired using methanol-*d*_4_ (CD_3_OD-*d*_4_). The ^1^H NMR spectrum of compound **4** reveals the following: an alpha proton at δ_H_ 4.70; beta protons at δ_H_ 3.31 and 3.12 of dimethylallyl tryptophan; indole ring protons at δ_H_ 7.44, 7.11, 7.01, and 6.85; and a dimethylallyl group with protons at δ_H_ 5.36, 3.40 (2H), and 1.75 (6H). The ^13^C NMR and HSQC spectra indicated that compound **4** bears 10 olefinic carbons (δ_C_ 138.5, 136.3, 132.4, 127.1, 125.6, 123.8, 120.9, 119.0, 111.4, and 110.9), 2 carbonyl groups (δ_C_ 175.3 and 173.2), and 3 methyl groups (δ_C_ 25.9, 22.4, and 17.9). The COSY correlations between H-4 (δ_H_ 7.44) and H-5 (δ_H_ 6.85) and the HMBC correlations from H-4 to C-3 (δ_C_ 110.9), C-3a (δ_C_ 127.1), C-6 (δ_C_ 136.3), and C-7a (δ_C_ 138.5); from H-7 (δ_H_ 7.11) to C-3a and C-5 (δ_C_ 120.9); and from H-2 (δ_H_ 7.01) to C-3, C-3a and C-7a established the indole ring of **4**. The COSY correlation of H-9 (δ_H_ 4.70) with H_2_-8 (δ_H_ 3.31 and 3.12) and the HMBC correlations from H_2_-8 to C-10 (δ_C_ 175.3), C-9 (δ_C_ 54.8), C-3, C-3a, and C-2 (δ_C_ 123.8) indicated that **4** bears the tryptophan moiety. The characteristic signals for a dimethylallyl moiety were observed by the COSY correlation with H-1′ (δ_H_ 3.40) to H-2′ (δ_H_ 5.36) and the HMBC correlations from H-4′ (δ_H_ 1.75) and H-5′ (δ_H_ 1.75) to C-2′ (δ_C_ 125.6) and from H-4′, H-5′, and H-1′ to C-3′ (δ_C_ 132.4). Lastly, the dimethylallyl group and tryptophan were linked by the HMBC correlations from H-1′ to C-5, C-6, and C-7 (δ_C_ 111.4). An *N*-Acetyl group was confirmed by signals for carbonyl carbon (δ_C_ 173.2) and a methyl group (δ_H_ 1.90 and δ_C_ 22.4). The HMBC correlations from H-9 and H-12 (δ_H_ 1.90) to C-11 (δ_C_ 173.2) constructed the connections between these partial structures of **4** (Figure 2 and Figure 3). Thus, the planar structure of **4** was determined as *N*-Acetyl-6-dimethylallyltryptophan. The absolute configurations of the compounds were assigned by comparing experimental circular dichroism (CD) and calculated CD spectra (Figures S25 and S26) and the optical rotation comparison with the reference [19,22].

### 2.3. Assessment of Antifungal Efficacy

To evaluate the antifungal efficacy, we performed a minimum inhibitory concentration (MIC) assay to assess the effectiveness of the compounds against major invasive fungal pathogens, including *Candida albicans*, *C. glabrata*, *C. auris*, and *Cryptococcus neoformans*. Notably, compound **1** demonstrated broad-spectrum inhibitory efficacy with an IC_50_ of 0.043 mM against *C. glabrata* and 0.35 mM against *C. albicans*, *C. auris*, and *C. neoformans*. In contrast, compounds **2**, **3**, and **4** did not exhibit significant inhibitory effects on any of the tested fungal pathogens (Table 5).

**Figure 4:**
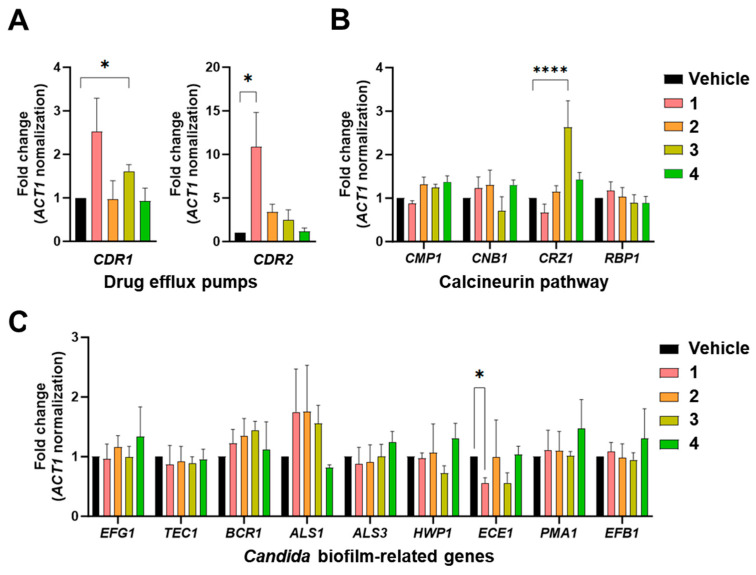
Gene expression regulated by compounds in *Candida albicans*. *Candida albicans* cells in early exponential growth phase were cultured in yeast-extracted peptone medium and treated with drugs at the IC_50_ level. Total RNA was extracted, converted into cDNA, and subsequently analyzed using quantitative PCR. (**A**) Genes encoding drug efflux pumps. (**B**) Genes involved in calcineurin signaling pathway. (**C**) Genes associated with biofilm formation. The *p* values from the multiple comparisons in ANOVA analysis are as follows: *, *p* < 0.05; ****, *p* < 0.0001.

To further understand the molecular impact of these compounds, we examined the changes in gene expression after treatment. Drug efflux pumps, such as *CDR1* and *CDR2*, which are commonly upregulated in response to antifungal agents, were analyzed [23]. Our results revealed that compound **1** significantly induced *CDR2* expression, while compound **3** led to a significant increase in *CDR1* expression (Figure 4A). Additionally, with respect to cell wall integrity, compound **3** induced the expression of *CRZ1*, a transcription factor in the calcineurin signaling pathway (Figure 4B). Interestingly, compound **1** treatment led to the repression of *ECE1*, the gene responsible for producing candidalysin, a key virulence factor in *C. albicans* (Figure 4C). This gene repression suggested that compound **1** could be a potential therapeutic agent for targeting virulence mechanisms. Based on these findings, we selected compounds **1** and **3** for further analysis because of their drug-like effects on *C. albicans*.

### 2.4. Optimization of Antifungal Potency

**Figure 5:**
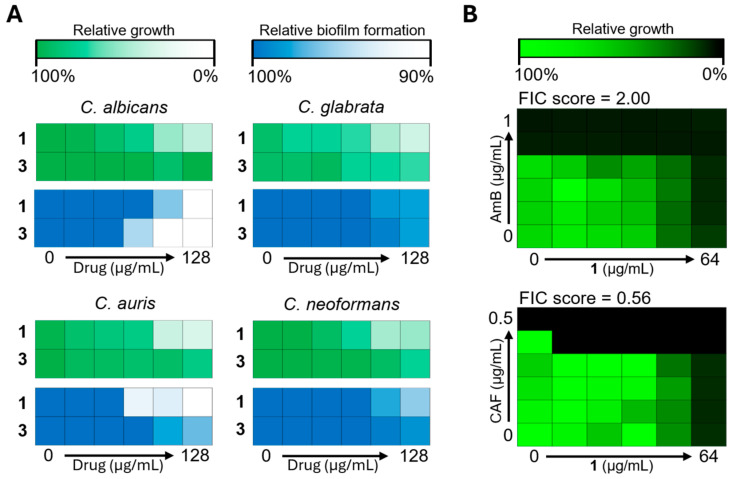
Evaluation of biofilm inhibition efficacy and drug synergy in *Candida albicans*. (**A**) Fungal growth following treatment with compounds **1** and **3** was measured by absorbance at OD 600 nm. After washing, biofilm formation was stained with crystal violet, and the residual dye was destained and measured by absorbance at OD 590 nm. (**B**) Compound **1** was tested in combination with amphotericin B (AmB) and caspofungin (CAF) using a checkerboard assay to assess drug synergy.

We examined and compared the inhibition of fungal growth and biofilm formation by compounds **1** and **3**. The growth inhibition efficacy of compound **1** was closely correlated with biofilm reduction. In *C. albicans* and *C. auris*, which experienced up to 50% growth inhibition by compound **1**, biofilm production was reduced by up to 10%. Compound **3** inhibited the growth of *C. glabrata* by up to 5% and displayed a similar pattern of biofilm inhibition. Interestingly, despite the lower efficacy of compound **3** in growth inhibition, *C. albicans* biofilms were reduced by up to 10% and *C. auris* biofilms by up to 6% (Figure 5A). Compound **1** exhibited broad-spectrum growth inhibition against fungal pathogens such as *C. albicans*, *C. glabrata*, *C. auris*, and *C. neoformans*, whereas compound **3** effectively inhibited biofilm formation in *C. albicans* and *C. auris*.

Subsequently, we investigated the potential synergistic effects of compound **1** in combination with standard antifungal drugs to enhance its growth inhibitory efficacy against *C. albicans* (Figure 5B). The combination of compound **1** with amphotericin B showed no synergistic effect (FIC score = 2.00; no interaction). However, caspofungin exhibited an additive interaction with the two agents (FIC score = 0.56). These findings suggest that, although compound **1** exhibits broad-spectrum antifungal activity, particularly against *C. albicans*, its combination with caspofungin offers an additive therapeutic effect, indicating its potential for enhanced treatment strategies targeting fungal pathogens.

## 3. Materials and Methods

### 3.1. General Experimental Procedures

General rotations were measured using a Jasco P-2000 polarimeter (Tokyo, Japan) with a 1.0 cm cell. UV spectra and LC/MS data were recorded using an Agilent G6125B MSD system coupled with an Agilent Technologies 1260 series Infinity II LC system (Agilent Technologies, Santa Clara, CA, USA) using a reversed-phase Phenomenex Luna C18 column (Phenomenex, Torrance, CA, USA, 100 × 4.6 mm, 5 µm). CD spectra were obtained with a 1 mm cell using Chirascan Plus (Applied Photophysics, Leatherhead, Surrey, UK). Infrared (IR) spectra were acquired using a Spectrum 3 Fourier Transform Infrared Spectrometer (PerkinElmer, Waltham, MA, USA). High-resolution electrospray ionization mass spectra (HR-ESI-MS) were obtained using an Agilent Technologies 1290 series HPLC coupled with an Agilent 6530 iFunnel Q-TOF LC/MS system (Agilent Technologies, Santa Clara, CA, USA). Moreover, ^1^H, ^13^C, and 2D NMR spectra were recorded on an Agilent VNMRS 600 MHz spectrometer (Agilent Technologies, Santa Clara, CA, USA) at the Korea Basic Science Institute (KBSI) in Gwangju and a Bruker Advance III 700 MHz spectrometer (Bruker, Billerica, MA, USA) and a Bruker Advance II 900 MHz spectrometer (Bruker, Billerica, MA, USA) at the KBSI in Ochang. HPLC purification was performed on a Waters system (Waters, Milford, MA, USA) 1525 binary HPLC pump and 996 photodiode array detector with a YMC (YMC, Kyoto, Japan) Pack-ODS-A-C18 column (250 × 10 mm, 5 μm).

### 3.2. Collection of Tidal Flat Samples and Isolation of Bacterial Strain

Tidal mudflat sediment samples were collected in Muan, Republic of Korea, in August 2020. Mixtures of dried samples and 4 mL of sterilized water (2 g each) were heated to 55 °C and sonicated. The mixtures were spread on A1 medium (agar 18 g, sea salt 33 g, and 1 L of distilled water), Chitin medium (chitin 4 g, K_2_HPO_4_ 0.75 g, MgSO_4_∙7H_2_O 0.5 g, KH_2_PO_4_ 3.5 g, FeSO_4_∙7H_2_O 10 mg, MnCl_2_∙7H_2_O 10 mg, ZnSO_4_∙7H_2_O 10 mg, agar 18 g, sea salt 33 g, and 1 L of distilled water), SIM medium (casein 0.4 g, starch 1 g, KNO_3_ 0.5 g, K_2_HPO_4_ 0.2 g, MgSO_4_∙7H_2_O 0.1 g, CaCO_3_ 0.1 g, agar 18 g, sea salt 33 g, and 1 L of distilled water), TWYE medium (K_2_HPO_4_ 0.5 g, yeast extract 0.25 g, agar 18 g, sea salt 33 g, and 1 L of distilled water), Kuster medium (glycerol 10 mL, casein 0.3 g, KNO_3_ 3 g, K_2_HPO_4_ 2 g, NaCl 2 g, MgSO_4_∙7H_2_O 0.05 g, CaCO_3_ 20 mg, FeSO_4_ 10 mg, agar 18 g, sea salt 33 g, and 1 L of distilled water) with cycloheximide (50 mg/L), and nalidixic acid (20 mg/L). The CMS18 strain was isolated from the TWYE agar medium and identified as *Saccharomonospora* sp. (99.9% identical to *Saccharomonospora azurea*) based on 16S rDNA gene sequence analysis (GenBank accession number AGIU02000033.1).

### 3.3. DNA Extraction for Metagenomics

DNA was prepared using the FastDNA^®^ Spin Kit for soil (MP Bio-medicals, Irvine, CA, USA), and its concentration was determined using an EpochTM Spectrometer (BioTek, Winooski, VT, USA). The hypervariable regions V3 and V4 of the 16S rRNA gene of bacteria were amplified by using the primer pair 341F [5′-TCGTCGGCAGCGTC-AGATGTGTATAAGAGACAG-CCTACGGGNGGCWGCAG-3′, 50mer] and 805R [5′-GTCTCGTGGGCTCGG-AGATGTGTATAAGAGACAG-GACTACHVGGGTATCTAATCC-3′, 55mer] followed by Illumina’s 16S Metagenomics Protocol (Illumina, Inc., San Diego, CA, USA) for the first amplicon PCR. The second PCR (index PCR) was conducted with the forward index i5 [AATGATACGGCGACCACCGAGATCTACAC-XXXXXX-TCGTCGGCAGCGTC, composition: left–i5 (XXXXXXXX)–right, 51mer] and reverse index i7 [CAAGCAGAAGACGGCATACGAGAT-GTCTCGTGGGCTCGG, composition: left–i5 (XXXXXXXX)–right, 47mer]. The PCR reagents included 10× buffer (2.5 L), dNTP (2.5 L), forward primer (10 pmol/L, 1 L), and reverse primer (10 pmol/L, 1 L). Taq polymerase (TaKaRa Ex Taq DNA polymerase, 1000 U), DNA (2 L), and D.W. (15.75 L) were used. The total volume of the PCR reaction was 25 µL. The Quanti-iT PicoGreen dsDNA Assay Kit provided by Invitrogen (Invitrogen, Carlsbad, CA, USA) in conjunction with the Agilent 2100 Bioanalyzer System was utilized in order to ascertain both the quantity and quality of a single library (Agilent Technologies, Santa Clara, CA, USA). A MiSeq^®^ Reagent Kit version 2 was used to perform paired-end sequencing on the samples (500 cycles; Illumina, Inc., San Diego, CA, USA).

### 3.4. Bioinformatics Analysis

To determine Q-values by sequence, sequences that could potentially be misinterpreted as “new species” based on their Q-value were eliminated. Trimmomatic software (version 0.40) was used to analyze next-generation sequencing (NGS) data produced by Illumina, and the researchers independently developed the code to evaluate other NGS data. The Pandaseq software (version 2.11) was implemented for bioinformatics purposes; however, this was not the case for PacBio single-end reads or CCS data [24]. In conjunction with in-house algorithms, the CJ Bioscience workflow was employed to eliminate the 16S rRNA PCR primer sequences using the in-house EzBioCloud 16S rRNA database. The annealing procedure generated these primer regions; therefore, their elimination was necessary. The 16S rRNA sequences were matched using various reference databases. This contributes to a reduction in computational labor and an improvement in accuracy. For additional analysis, both in-house methods and the HMMER program were used. Approximately 0.5% of raw NGS data contained sequencing errors that may have occurred at any time. Since the same gene was sequenced several times during the NGS studies, these mistakes could be corrected by applying suitable models. To determine the identity of each sequence, the pipeline relied on the tried-and-true identification techniques stored in EzBioCloud. The first phase of this two-part technique involved searching reference databases using the USEARCH program. The second step was to calculate similarity using a robust pairwise alignment. Query sequences were considered to be at the species level if they were at least 97% similar to the reference sequences in EzBioCloud. Lower cutoffs for sequence similarity were employed for higher taxonomic levels, such as genera. *x = the degree to which the sequence in question is comparable to that of a reference sequence: species (×97%), genus (97 > ×94.5%), family (94.5 > ×86.5%), order (86.5 > ×82%), class (82 > ×78.5%), and phylum (78.5 > ×75%). In this investigation, the cutoff values determined by Yarza et al. were taken into consideration [25]. However, during the process of detecting chimeric sequences, sequences with less than 97% similarity in the identification phase were identified. Because this sequence had already been identified at the species level, it was eliminated as the detection target. Operational taxonomic units (OTUs) are arbitrary classification units frequently referred to as a “species” in microbial community research. OTUs are operational taxonomic units.

### 3.5. Taxonomic Analysis and Alpha- and Beta-Diversity

Each sequence was partitioned into multiple operational OTUs using various methods and computer programs. Sequences that shared less than 97% similarity were defined as a species, and each was assigned an OTU, with the exception of chimeras. Using the CD-hit and UCLUST algorithms, cluster analysis was conducted on sequences that were less than 97% identical, and an OTU was established for each cluster that emerged. The final OTU information was obtained by integrating the OTUs discovered in steps one and two. The OTU produced as a single sequence was omitted from the diversity analysis because its existence led to a greater increase in the diversity index than the real value. In addition to the Shannon and Simpson statistics, various indices of species abundance and evenness were included to calculate alpha diversity.

### 3.6. Large-Scale Cultivation and Extraction

The CMS18 strain was cultured in 50 mL of YEME medium (glucose 10 g, yeast extract 3 g, malt extract 3 g, peptone 5 g, soybean extract 2 g, sea salt 33 g, and 1 L of distilled water) in a 125 mL flask on a rotary shaker for 3 days at 30 °C and 190 rpm. For the first scale-up, a 3.5 mL aliquot of the broth culture was used to inoculate 150 mL of YEME medium in a 500 mL flask, which was then cultured under the same conditions. Twenty milliliters of the culture was transferred to 1 L of YEME medium in 2.5 L ultra-yield flasks (10 each 1 L, total volume 10 L) for 5 days under the same fermentation conditions. The CMS18 strain was isolated from the whole culture (in EtOAc) using 15 L of ethyl acetate. The EtOAc and water layers were separated, and the remaining water in the EtOAc layer was eliminated by adding anhydrous sodium sulfate. The EtOAc extract was concentrated under vacuum to obtain a solid substance.

### 3.7. Isolation and Purification of Compounds

The CMS18 strain dried material was fractionated over a C_18_ reversed-phase column (YMC ODS-A C_18_, 50 μm silica gel) using a step gradient solvent system (20, 40, 60, 80, and 100% MeOH/H_2_O). Compound **1** was detected in the CMS18 80% MeOH/H_2_O fraction, and compounds **2**, **3**, and **4** were detected in the CMS18 60% MeOH/H_2_O fraction. Each fraction was filtered through a syringe filter (Advantec, Tokyo, Japan, HP020AN) and subjected to semi-preparative reversed-phase HPLC at a flow rate of 2 mL/min using a linear gradient and an isocratic solvent system. The CMS18 80% fraction was purified using isocratic 70% CH_3_CN/H_2_O, and **1** was eluted after 24.6 min. The CMS18 60% fraction was purified using a linear gradient of 25% to 40% CH_3_CN/H_2_O for 40 min (YMC ODS-A-C18:250 × 10 mm, 5 µm, UV detection at 254 nm). Under these HPLC conditions, **2** was eluted at 17 min, **3** at 30, and **4** at 33 min. Finally, the samples were purified to yield the pure compounds **1** (2.1 mg), **2** (10 mg), **3** (1.7 mg), and **4** (2 mg).

*6-dimetyhlallyl-indole* (**1**): colorless oil, UV (MeOH) *λ*_max_ (log ε) 225 (3.78) nm, 280 (3.44) nm; IR (ATR) *ν*_max_ 3116, 3050, 1534, 1350 cm^−1^; and ^1^H and ^13^C NMR data (Table 1), HR-ESI-MS *m*/*z* 186.1277 [M + H]^+^ (calculated for C_13_H_16_N, 186.1277 [M + H]^+^).

*6-dimethylallyl-l-tryptophan* (**2**): yellow oil, [α${]}_{25}^{D}$ + 2.49 (c 0.1, MeOH); UV (MeOH) *λ*_max_ (log ε) 225 (3.98) nm, 280 (3.40) nm; IR (ATR) *ν*_max_ 3174, 3059, 1804, 1268 cm^−1^; CD (c 0.01, MeOH) (Δε) 228 (2.65), 260 (−0.73); and ^1^H and ^13^C NMR data (Table 2), HR-ESI-MS *m*/*z* 273.1604 [M + H]^+^ (calculated for C_16_H_21_N_2_O_2_, 273.1597 [M + H]^+^).

*penipaline D* (**3**): yellow oil, [α${]}_{25}^{D}$ −1.15 (c 0.1, MeOH); UV (MeOH) *λ*_max_ (log ε) 225 (4.02) nm, 270 (3.25) nm; IR (ATR) *ν*_max_ 3224, 3049, 2904, 1287 cm^−1^; and ^1^H and ^13^C NMR data (Table 3), HR-ESI-MS *m*/*z* 285.1588 [M + H]^+^ (calculated for C_17_H_21_N_2_O_2_, *m*/*z* 285.1597 [M + H]^+^).

*N-acetyl-6-dimethylallyl-l-tryptophan* (**4**): colorless oil, [α${]}_{25}^{D}$ +2.82 (c 0.1, MeOH); UV (MeOH) *λ*_max_ (log ε) 225 (4.12) nm, 280 (3.52) nm; IR (ATR) *ν*_max_ 3443, 3069, 1786, 1414, 1241, 1128 cm^−1^; CD (c 0.01, MeOH) (Δε) 230 (2.01), 268 (−0.98); and ^1^H and ^13^C NMR data (Table 4), HR-ESI-MS *m*/*z* 315.1713 [M + H]^+^ (cacld for C_18_H_23_N_2_O_3_, *m*/*z* 315.1703 [M + H]^+^).

### 3.8. Strains and Growth Conditions for Bioactivity Test

*Candida albicans* SC5314, *C. glabrata* BG2, *C. auris* B8441, and *C. neoformans* H99 strains were grown in a 35 °C incubator and sub-cultured in yeast-extracted peptone dextrose (YPD) broth to perform the experiments.

### 3.9. Minimal Inhibitory Concentration (MIC) Assay

We conducted MIC assays to determine the efficacy of the compounds against fungal pathogens, following the EUCAST guidelines [26,27]. Overnight cultured cells were spun down and washed three times with phosphate-buffered saline (PBS), and the cell number was synchronized with an OD_600_ value of 1.0 as a fungal cell stock solution. A final 1/100 dilution in RPMI 1640 medium (#R6504; Sigma-Aldrich, St. Louis, MO, USA) was added to 96-well plates for the assay. Each compound was dissolved in dimethyl sulfoxide (DMSO) to a final concentration of 27.7 mM (**1**), 18.8 mM (**2**), 18.0 mM (**3**), and 16.3 mM (**4**) as the drug stock solution. For the assay, 5 μL of the drug stock was inoculated into 195 µL of the fungal cell stock solution and serially diluted into the 100 µL of the fungal cell stock solution for 2-fold dilution. The final drug concentration for the MIC assay ranged from 5.4 μM to 0.69 mM (**1**), 3.7 μM to 0.47 mM (**2**), 3.5 μM to 0.45 mM (**3**), and 3.2 μM to 0.41 mM (**4**). The plates were incubated at 35 °C in a standing incubator for 48 h, and the OD_600_ value was detected by a microplate reader.

### 3.10. Gene Expression Analysis

*C. albicans* was treated with compounds, followed by analysis of gene expression, particularly of genes related to drug responses and virulence factors. Overnight-grown cells were inoculated into the fresh YPD broth at an OD_600_ value of 0.2 and sub-cultured to 0.8 in the early exponential growth phage. Each compound was added to the cultured broth at a 50% inhibitory concentration in Table 5, with a maximum concentration of 0.69 mM (**1**), 0.47 mM (**2**), 0.45 mM (**3**), and 0.41 mM (**4**). After 3 h of treatment, the cells were centrifuged, and total RNA was extracted using RNAiso PLUS (TaKaRa #9108) with bead beating. The extracted RNA was used as a template to synthesize cDNA, and its expression was detected using real-time quantitative PCR (TaKaRa, Kusatsu, Shiga, Japan, CronoSTAR 96 Real-Time PCR System).

### 3.11. ECD Calculation

The ground-state geometries were computed with density functional theory (DFT) calculations using Turbomole 7.7.1, the basis set def-SVP for all atoms, and the B3LYP/DFT functional level. The ground states were further confirmed using a harmonic frequency calculation. The calculated ECD data corresponding to the optimized structures were acquired with TD-DFT at the B3LYP/DFT functional level using the basis set def-TZVPP for all atoms. The CD spectra were stimulated by overlapping for each transition according to the equation below, where *σ* is the width of the band at the height of 1/*e* and Δ*E_i_* and *R_i_* are the excitation energies and rotatory strengths for transition *i*, respectively. In this calculation, the value of *σ* was taken to be 0.10 eV.

$$∆\epsilon \left(E\right)=\frac{1}{2.297\times 10^{-39}}\frac{1}{\sqrt{2\pi \sigma }}\sum_{i}^{A}\Delta E_{i}R_{i}e^{\left[-(E-∆E_{i}{)}^{2}/(2\sigma {)}^{2}\right]}$$

## Figures and Tables

**Table 1:** ^1^H and ^13^C NMR spectroscopic data of 6-dimethylallyl-indole (**1**) in CD_3_OD-*d*_4_.

	6-Dimethylallyl-indole (1)	
Position	δ_C_, Type	δ_H_, Mult (*J* in Hz)
2	124.9, CH	7.14, d (3.0)
3	102.0, CH	6.36, d (3.0)
3a	127.6, C	-
4	120.9, CH	7.42, d (8.0)
5	121.1, CH	6.83, d (8.0)
6	136.0, C	-
7	111.2, CH	7.16, s
7a	138.1, C	-
1′	3.52, CH_2_	3.41, d (7.5)
2′	125.6, CH	5.38, t (7.5)
3′	132.3, C	-
4′	17.9, CH_3_	1.76, s
5′	25.9, CH_3_	1.76, s

^1^H and ^13^C NMR data were recorded at 700 and 175 MHz.

**Table 2:** ^1^H and ^13^C NMR spectroscopic data of 6-dimethylallyl-l-tryptophan (**2**) in CD_3_OD-*d*_4_.

	6-Dimethylallyl-l-tryptophan (2)	
Position	δ_C_, Type	δ_H_, Mult (*J* in Hz)
2	124.9, CH	7.14, s
3	108.4, C	-
3a	126.5, C	-
4	118.9, CH	7.52, d (8.0)
5	121.4, CH	6.89, d (8.0)
6	136.9, C	-
7	111.7, CH	7.17, s
7a	138.8, C	-
8	28.0, CH_2_	3.49, dd (15.0, 5.0)
		3.23, dd (15.0, 8.0)
9	55.5, CH	4.07, dd (8.0, 5.0)
10	173.4, C	-
1′	35.5, CH_2_	3.41, d (7.5)
2′	125.4, CH	5.35, t (7.5)
3′	132.6, C	-
4′	17.9, CH_3_	1.74, s
5′	25.9, CH_3_	1.74, s

^1^H and ^13^C NMR data were recorded at 600 and 150 MHz.

**Table 3:** ^1^H and ^13^C NMR spectroscopic data of penipaline D (**3**) in CD_3_OD-*d*_4_.

	Penipaline D (3) ^a^	
Position	δ_C_, Type	δ_H_, Mult (*J* in Hz)
1	41.9, CH_2_	4.39, d (6.5)
3	59.0, CH	3.94, m
4	24.2, CH_2_	3.41, m
		3.03, m
5	107.7, C	-
6	125.8, C	-
7	118.8, CH	7.36, d (8.0)
8	121.5, CH	6.89, d (8.0)
9	137.3, C	-
10	111.4, CH	7.11, s
11	138.9, C	-
13	126.1, C	-
14	173.9, C	-
1′	35.5, CH_2_	3.41, m
2′	125.3, CH	5.36, t (7.5)
3′	132.7, C	-
4′	17.9, CH_3_	1.75, s
5′	25.9, CH_3_	1.75, s

^a 1^H and ^13^C NMR data were recorded at 700 and 175 MHz.

**Table 4:** ^1^H and ^13^C NMR spectroscopic data of *N*-acetyl-6-dimethylallyl-l-tryptophan (**4**) in CD_3_OD-*d*_4_.

	*N*-Acetyl-6-dimethylallyl-l-tryptophan (4) ^c^	
Position	δ_C_, Type	δ_H_, Mult (*J* in Hz)
2	123.8, CH	7.01, s
3	110.9, C	4.70, dd (8.0, 5.0)
3a	127.1, C	3.31, m
4	119.0, CH	7.44, d (8.0)
5	120.9, CH	6.85, d (8.0)
6	136.3, C	-
7	111.4, CH	7.11, s
7a	138.5, C	-
8	28.6, CH_2_	3.31, m
		3.12, dd (15.0, 8.0)
9	54.8, CH	4.70, dd (8.0, 5.0)
10	175.3, C	-
11	173.2, C	-
12	22.4, CH_3_	1.90, s
1′	123.8, CH	7.01, s
2′	173.2, C	-
3′	22.4, CH_3_	1.90, s
4′	35.5, CH_2_	3.40, d (7.5)
5′	125.6, CH	5.36, t (7.5)

^c 1^H and ^13^C NMR data were recorded at 900 and 225 MHz.

**Table 5:** Antifungal activity of the compounds against human fungal pathogens.

	*C. albicans*			*C. glabrata*			*C. auris*			*C. neoformans*		
Cpd.	IC_20_	IC_50_	IC_90_	IC_20_	IC_50_	IC_90_	IC_20_	IC_50_	IC_90_	IC_20_	IC_50_	IC_90_
1	0.17	0.35	>0.69	0.04	0.04	>0.69	0.17	0.35	>0.69	0.17	0.35	>0.69
2	0.47	>0.47	>0.47	0.12	>0.47	>0.47	>0.47	>0.47	>0.47	0.47	>0.47	>0.47
3	>0.45	>0.45	>0.45	0.12	0.45	>0.45	0.45	>0.45	>0.45	0.45	>0.45	>0.45
4	>0.41	>0.41	>0.41	0.10	>0.41	>0.41	>0.41	>0.41	>0.41	>0.41	>0.41	>0.41

IC_20_, IC_50_, and IC_90_ refer to 20%, 50%, and 90% inhibitory concentrations (mM), respectively.

## Data Availability

The original data presented in the study are included in the article/Supplementary Material; further inquiries can be directed to the corresponding author.

## Supplementary Materials

The following supporting information can be downloaded from https://www.mdpi.com/article/10.3390/md22120539/s1, Figure S1. ^1^H NMR spectrum of 6-dimethylallyl-indole (**1**) at 700 MHz in CD_3_OD-*d*_4_; Figure S2. ^13^C NMR spectrum of 6-dimethylallyl-indole (**1**) at 175 MHz in CD_3_OD-*d*_4_; Figure S3. COSY NMR spectrum of 6-dimethylallyl-indole (**1**) at 700 MHz in CD_3_OD-*d*_4_; Figure S4. HSQC NMR spectrum of 6-dimethylallyl-indole (**1**) at 700 MHz in CD_3_OD-*d*_4_; Figure S5. HMBC NMR spectrum of 6-dimethylallyl-indole (**1**) at 700 MHz in CD_3_OD-*d*_4_; Figure S6. ^1^H NMR spectrum of 6-dimethylallyl-l-tryptophan (**2**) at 600 MHz in CD_3_OD-*d*_4_; Figure S7. ^13^C NMR spectrum of 6-dimethylallyl-l-tryptophan (**2**) at 150 MHz in CD_3_OD-*d*_4_; Figure S8. COSY NMR spectrum of 6-dimethylallyl-l-tryptophan (**2**) at 600 MHz in CD_3_OD-*d*_4_; Figure S9. HSQC NMR spectrum of 6-dimethylallyl-l-tryptophan (**2**) at 600 MHz in CD_3_OD-*d*_4_; Figure S10. HMBC NMR spectrum of 6-dimethylallyl-l-tryptophan (**2**) at 600 MHz in CD_3_OD-*d*_4_; Figure S11. ^1^H NMR spectrum of penipaline D (**3**) at 700 MHz in CD_3_OD-*d*_4_; Figure S12. ^13^C NMR spectrum of penipaline D (**3**) at 175 MHz in CD_3_OD-*d*_4_; Figure S13. COSY NMR spectrum of penipaline D (**3**) at 700 MHz in CD_3_OD-*d*_4_; Figure S14. HSQC NMR spectrum of penipaline D (**3**) at 700 MHz in CD_3_OD-*d*_4_; Figure S15. HMBC NMR spectrum of penipaline D (**3**) at 700 MHz in CD_3_OD-*d*_4_; Figure S16. ^1^H NMR spectrum of N-acetyl-6-dimethylallyl-l-tryptophan (**4**) at 900 MHz in CD_3_OD-*d*_4_; Figure S17. ^13^C NMR spectrum of *N*-acetyl-6-dimethylallyl-l-tryptophan (**4**) at 225 MHz in CD_3_OD-*d*_4_; Figure S18. COSY NMR spectrum of *N*-acetyl-6-dimethylallyl-l-tryptophan (**4**) at 900 MHz in CD_3_OD-*d*_4_; Figure S19. HSQC NMR spectrum of *N*-acetyl-6-dimethylallyl-l-tryptophan (**4**) at 900 MHz in CD_3_OD-*d*_4_; Figure S20. HMBC NMR spectrum of *N*-acetyl-6-dimethylallyl-l-tryptophan (**4**) at 900 MHz in CD_3_OD-*d*_4_; Figure S21. HR-ESI-MS data of 6-dimethylallyl-indole (**1**); Figure S22. HR-ESI-MS data of 6-dimethylallyl-l-tryptophan (**2**); Figure S23. HR-ESI-MS data of penipaline D (**3**); Figure S24. HR-ESI-MS data of N-acetyl-6-dimethylallyl-l-tryptophan (**4**); Figure S25. Experimental and calculated ECD spectra of (*R*)- and (*S*)-6-dimethylallyl-l-tryptophan (**2**); Figure S26. Experimental and calculated ECD spectra of (*R*)- and (*S*)-*N*-Acetyl-6-dimethylallyl-l-tryptophan (**4**); Table S1. ECD calculation of (*R*)- 6-dimethylallyl-l-tryptophan (**2**); Table S2. ECD calculation of (*S*)-6-dimethylallyl-l-tryptophan (**2**); Table S3. ECD calculation of (*R*)-*N*-Acetyl-6-dimethylallyl-l-tryptophan (**4**); Table S4. ECD calculation of (*S*)-*N*-Acetyl-6-dimethylallyl-l-tryptophan (**4**); Table S5. Cartesian coordinates of (*R*)-6-dimethylallyl-l-tryptophan (**2**); Table S6. Cartesian coordinates of (*S*)- 6-dimethylallyl-l-tryptophan (**2**); Table S7. Cartesian coordinates of (*R*)-*N*-Acetyl-6-dimethylallyl-l-tryptophan (**4**); Table S8. Cartesian coordinates of (*S*)-*N*-Acetyl-6-dimethylallyl-l-tryptophan (**4**).
