# Supplementary material for: Isolation and Characterization of Bioactive Compounds from Saccharomonospora sp. CMS18 and Their Antifungal Properties
Source: Mar Drugs. 2024 Nov 30;22(12):539. doi: 10.3390/md22120539 (PMC11677142; doi:10.3390/md22120539)
Supplement: Supplementary file 1 [file marinedrugs-22-00539-s001.zip › marinedrugs-3307388-supplementary.pdf]

Supporting Information

Isolation and Characterization of Bioactive Compounds from *Saccharomonospora* sp. CMS18 and Their Antifungal Properties

Soohyun Um[1,†], Hyeongju Jeong[2,†], Jeongwon Seo[2], Ji-Eun Park[3,4], Sang Heon Jung[5], Munhyung Bae[5], Kyung-Tae Lee[4,], and Kyuho Moon[6,7,*]*

[1]College of Pharmacy, Yonsei University, Incheon, 21983, Republic of Korea: soohyunum@yonsei.ac.kr (S.U.)

[2]College of Pharmacy, Research Institute of Pharmaceutical Sciences, Chonnam National University, Gwangju, 61186, Republic of Korea: 217843@jnu.ac.kr (H.J.); jeongwon.0522@gamil.com (J.S.)

[3]Korea Research Institute for Veterinary Biologics, Iksan, Jeonbuk State, Republic of Korea

[4]Korea Zoonosis Research Institute, Jeonbuk National University, Iksan, Jeonbuk State, Republic of Korea

[5]College of Pharmacy, Gachon University, Incheon 21936, Republic of Korea; fly6778@gachon.ac.kr (S.H.J.); baemoon89@gachon.ac.kr (M.B.)

[6]College of Pharmacy, Kyung Hee University, Seoul, 02447, Republic of Korea

[7]Department of Biomedical and Pharmaceutical Sciences, Graduate School, Kyung Hee University, Seoul, 02447, Republic of Korea

Corresponding author:

*(Lee, K-T) Tel: +82 E-mail: lee.kt@jbnu.ac.kr and (M, K) Tel: +82-2-961-5139. E-mail: kmoon@khu.ac.kr

Table of Contents

Figure S1. ^{1}H NMR spectrum of 6-dimethylallyl-indole (**1**) at 700 MHz in CD_3OD-d_4.

Figure S2. ^{13}C NMR spectrum of 6-dimethylallyl-indole (**1**) at 175 MHz in CD_3OD-d_4.

Figure S3. COSY NMR spectrum of 6-dimethylallyl-indole (**1**) at 700 MHz in CD_3OD-d_4.

Figure S4. HSQC NMR spectrum of 6-dimethylallyl-indole (**1**) at 700 MHz in CD_3OD-d_4.

Figure S5. HMBC NMR spectrum of 6-dimethylallyl-indole (**1**) at 700 MHz in CD_3OD-d_4.

Figure S6. ^{1}H NMR spectrum of 6-dimethylallyl-L-tryptophan (**2**) at 600 MHz in CD_3OD-d_4.

Figure S7. ^{13}C NMR spectrum of 6-dimethylallyl-L-tryptophan (**2**) at 150 MHz in CD_3OD-d_4.

Figure S8. COSY NMR spectrum of 6-dimethylallyl-L-tryptophan (**2**) at 600 MHz in CD_3OD-d_4.

Figure S9. HSQC NMR spectrum of 6-dimethylallyl-L-tryptophan (**2**) at 600 MHz in CD_3OD-d_4.

Figure S10. HMBC NMR spectrum of 6-dimethylallyl-L-tryptophan (**2**) at 600 MHz in CD_3OD-d_4.

Figure S11. ^{1}H NMR spectrum of penipaline D (**3**) at 700 MHz in CD_3OD-d_4.

Figure S12. ^{13}C NMR spectrum of penipaline D (**3**) at 175 MHz in CD_3OD-d_4.

Figure S13. COSY NMR spectrum of penipaline D (**3**) at 700 MHz in CD_3OD-d_4.

Figure S14. HSQC NMR spectrum of penipaline D (**3**) at 700 MHz in CD_3OD-d_4.

Figure S15. HMBC NMR spectrum of penipaline D (**3**) at 700 MHz in CD_3OD-d_4.

Figure S16. ^{1}H NMR spectrum of *N*-acetyl-6-dimethylallyl-L-tryptophan (**4**) at 900 MHz in CD_3OD-d_4.

Figure S17. ^{13}C NMR spectrum of *N*-acetyl-6-dimethylallyl-L-tryptophan (**4**) at 225 MHz in CD_3OD-d_4.

Figure S18. COSY NMR spectrum of *N*-acetyl-6-dimethylallyl-L-tryptophan (**4**) at 900 MHz in CD_3OD-d_4.

Figure S19. HSQC NMR spectrum of *N*-acetyl-6-dimethylallyl-L-tryptophan (**4**) at 900 MHz in CD_3OD-d_4.

Figure S20. HMBC NMR spectrum of *N*-acetyl-6-dimethylallyl-L-tryptophan (**4**) at 900 MHz in CD_3OD-d_4.

Figure S21. HR-ESI-MS data of 6-dimethylallyl-indole (**1**).

Figure S22. HR-ESI-MS data of 6-dimethylallyl-L-tryptophan (**2**).

Figure S23. HR-ESI-MS data of penipaline D (**3**).

Figure S24. HR-ESI-MS data of *N*-acetyl-6-dimethylallyl-L-tryptophan (**4**).

Figure S25. Experimental and calculated ECD spectra of (*R*)- and (*S*)-6-dimethylallyl-L-tryptophan (**2**).

Figure S26. Experimental and calculated ECD spectra of (*R*)- and (*S*)- *N*-Acetyl-6-dimethylallyl-L-tryptophan (**4**).

Table S1. ECD calculation of (*R*)- 6-dimethylallyl-L-tryptophan (**2**).

Table S2. ECD calculation of (*S*)- 6-dimethylallyl-L-tryptophan (**2**).

Table S3. ECD calculation of (*R*)-*N*-Acetyl-6-dimethylallyl-L-tryptophan (**4**).

Table S4. ECD calculation of (*S*)-*N*-Acetyl-6-dimethylallyl-L-tryptophan (**4**).

Table S5. Cartesian coordinates of (*R*)- 6-dimethylallyl-L-tryptophan (**2**).

Table S6. Cartesian coordinates of (*S*)- 6-dimethylallyl-L-tryptophan (**2**).

Table S7. Cartesian coordinates of (*R*)-*N*-Acetyl-6-dimethylallyl-L-tryptophan (**4**).

Table S8. Cartesian coordinates of (*S*)-*N*-Acetyl-6-dimethylallyl-L-tryptophan (**4**).

Figure S1. ^{1}H NMR spectrum of 6-dimethylallyl-indole (**1**) at 700 MHz in CD_3OD-d_4.

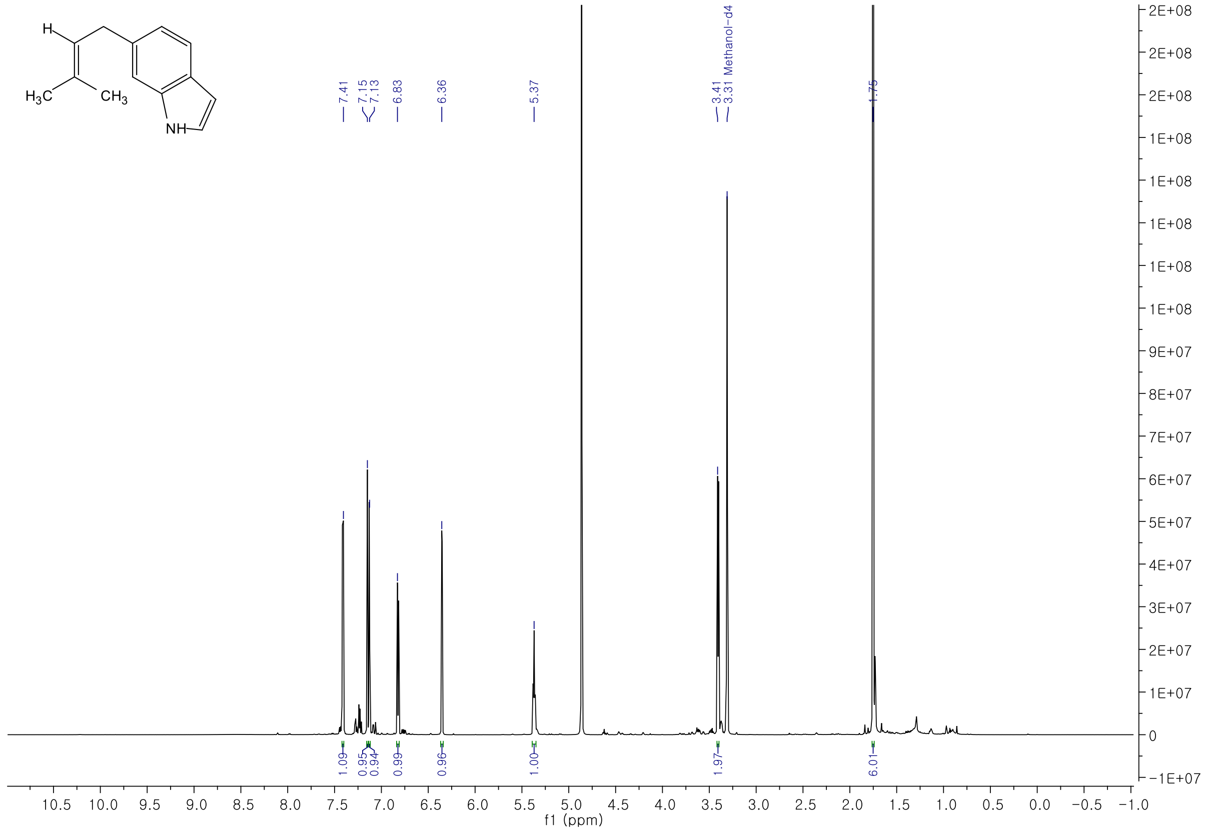

Figure S2. ^{13}C NMR spectrum of 6-dimethylallyl-indole (**1**) at 175 MHz in CD_3OD-d_4.

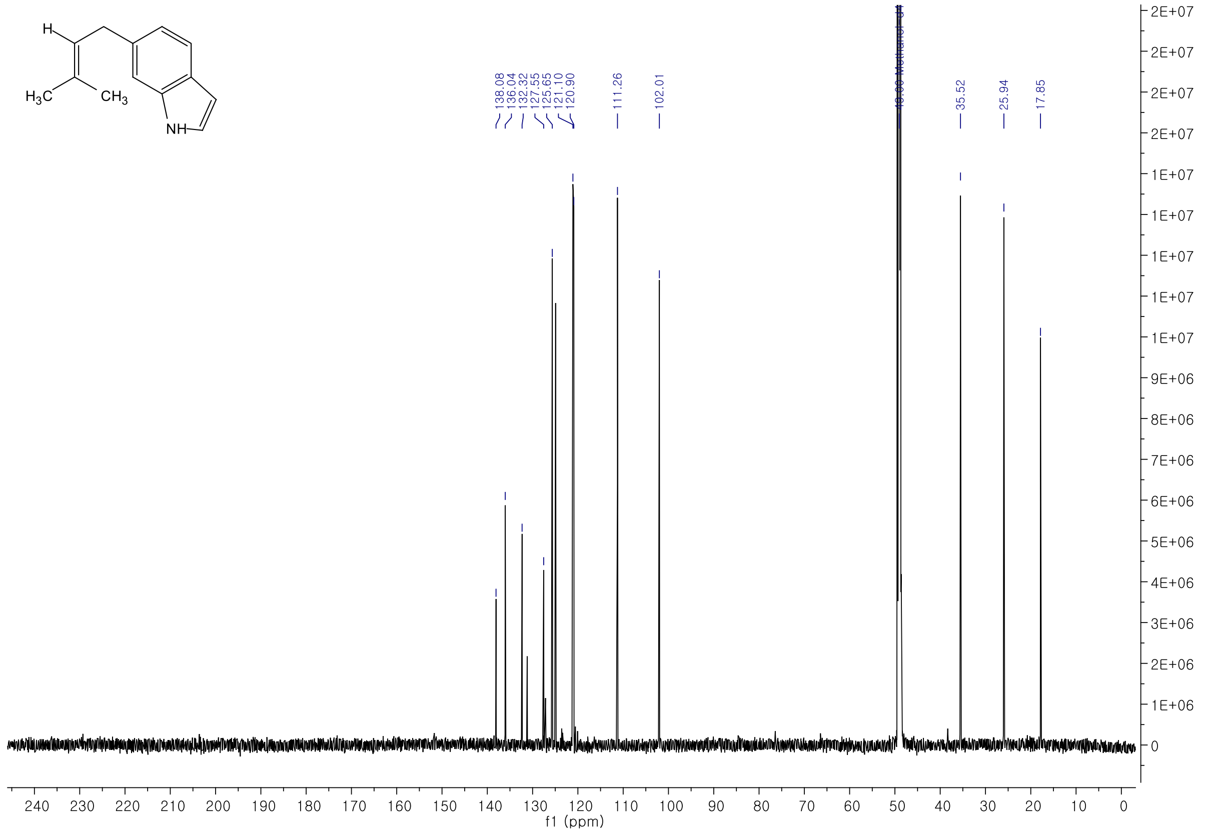

Figure S3. COSY NMR spectrum of 6-dimethylallyl-indole (**1**) at 700 MHz in CD_3OD-d_4.

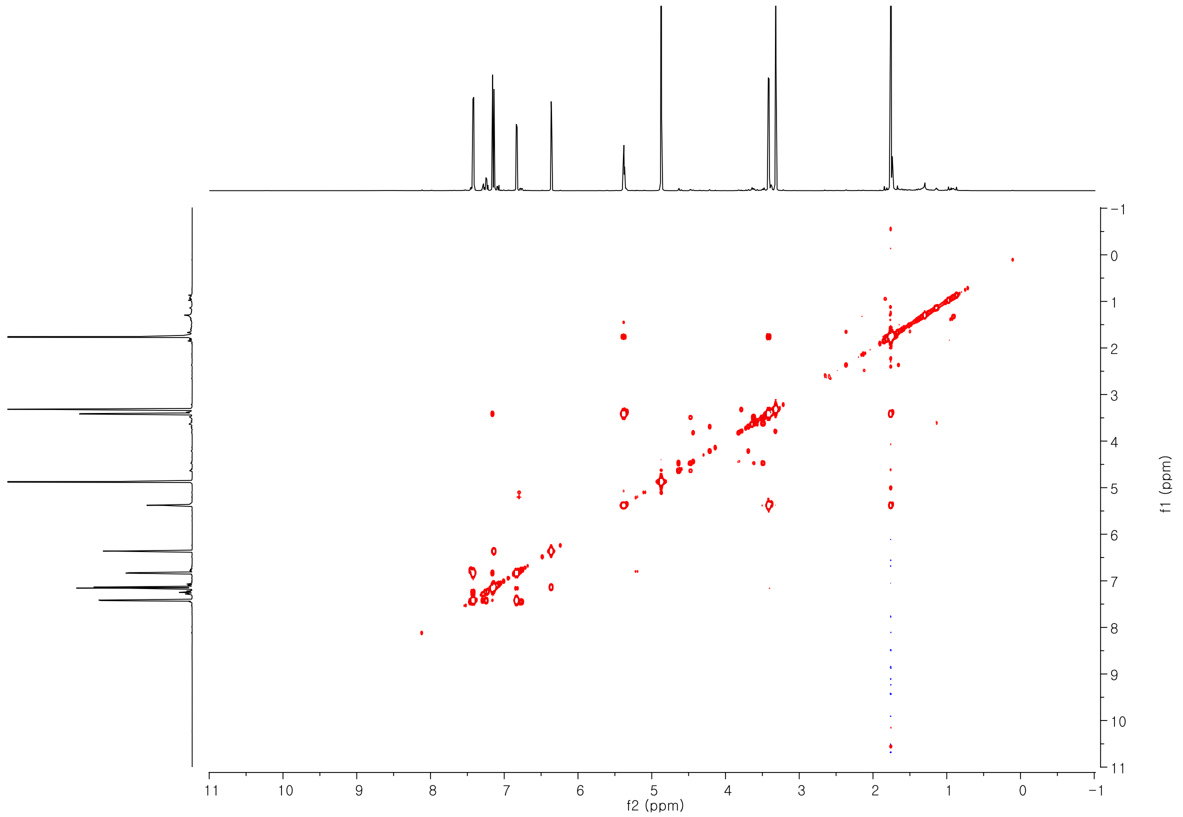

Figure S4. HSQC NMR spectrum of 6-dimethylallyl-indole (**1**) at 700 MHz in CD_3OD-d_4.

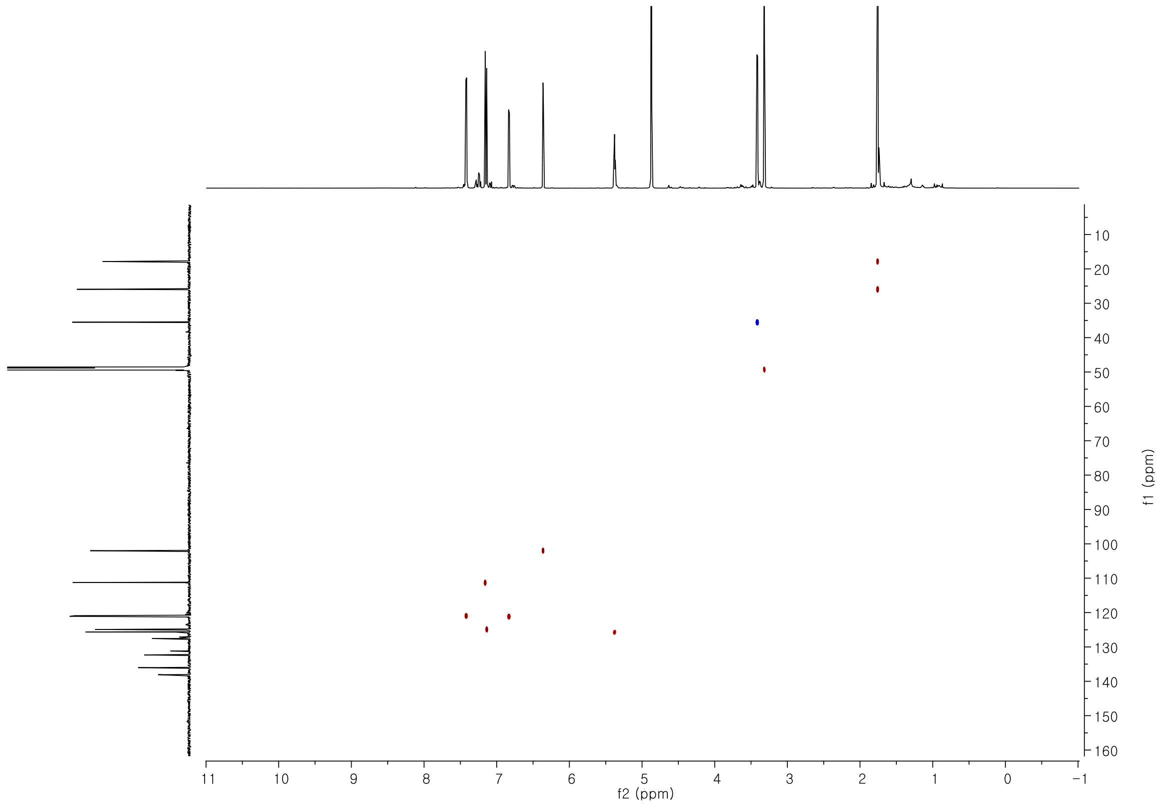

Figure S5. HMBC NMR spectrum of 6-dimethylallyl-indole (**1**) at 700 MHz in CD_3OD-d_4.

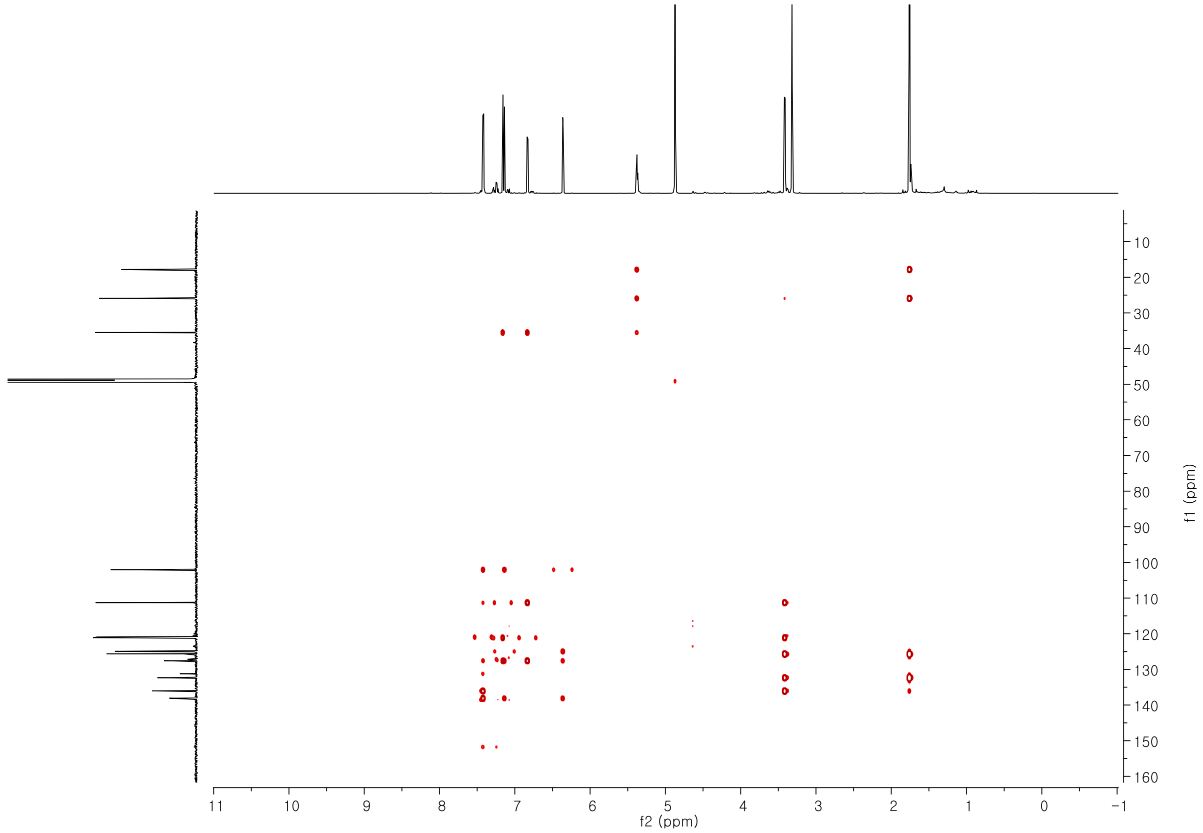

Figure S6. ^{1}H NMR spectrum of 6-dimethylallyl-L-tryptophan (**2**) at 600 MHz in CD_3OD-d_4.

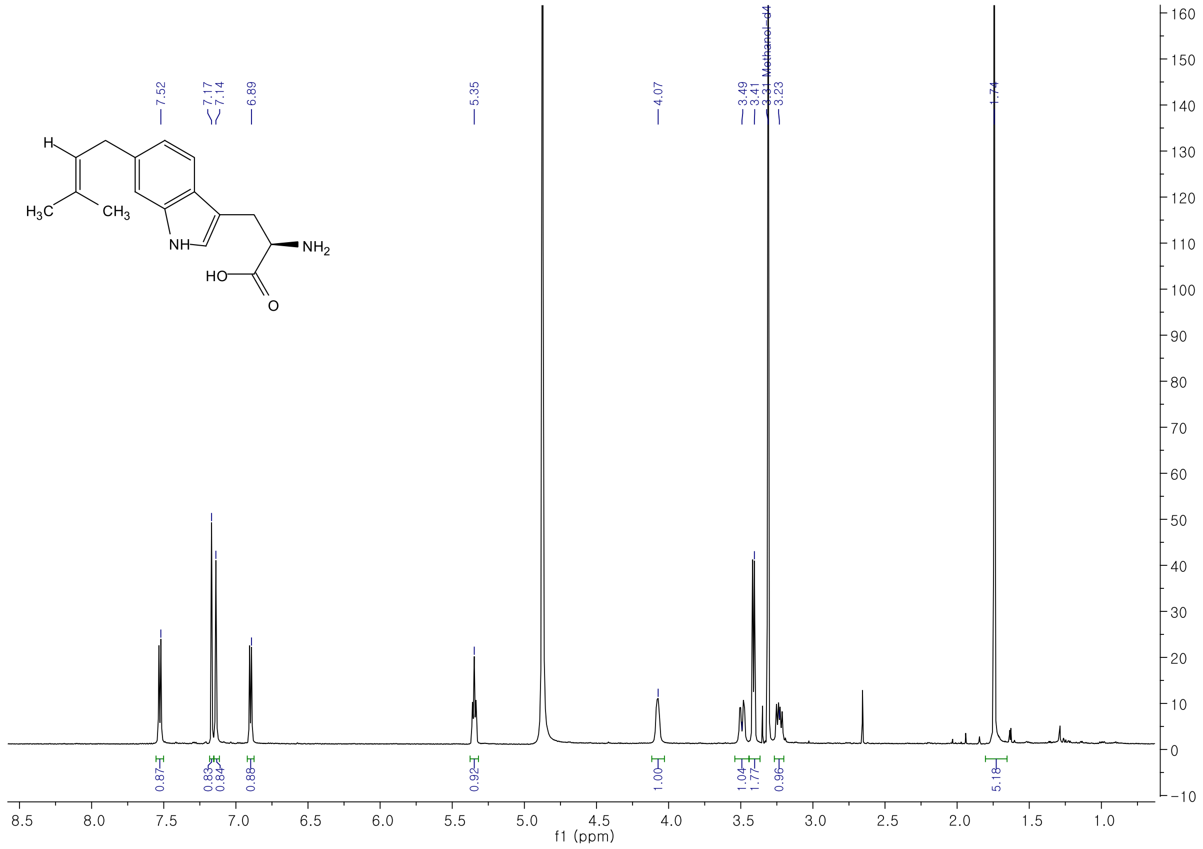

Figure S7. ^{13}C NMR spectrum of 6-dimethylallyl-L-tryptophan (**2**) at 150 MHz in CD_3OD-d_4.

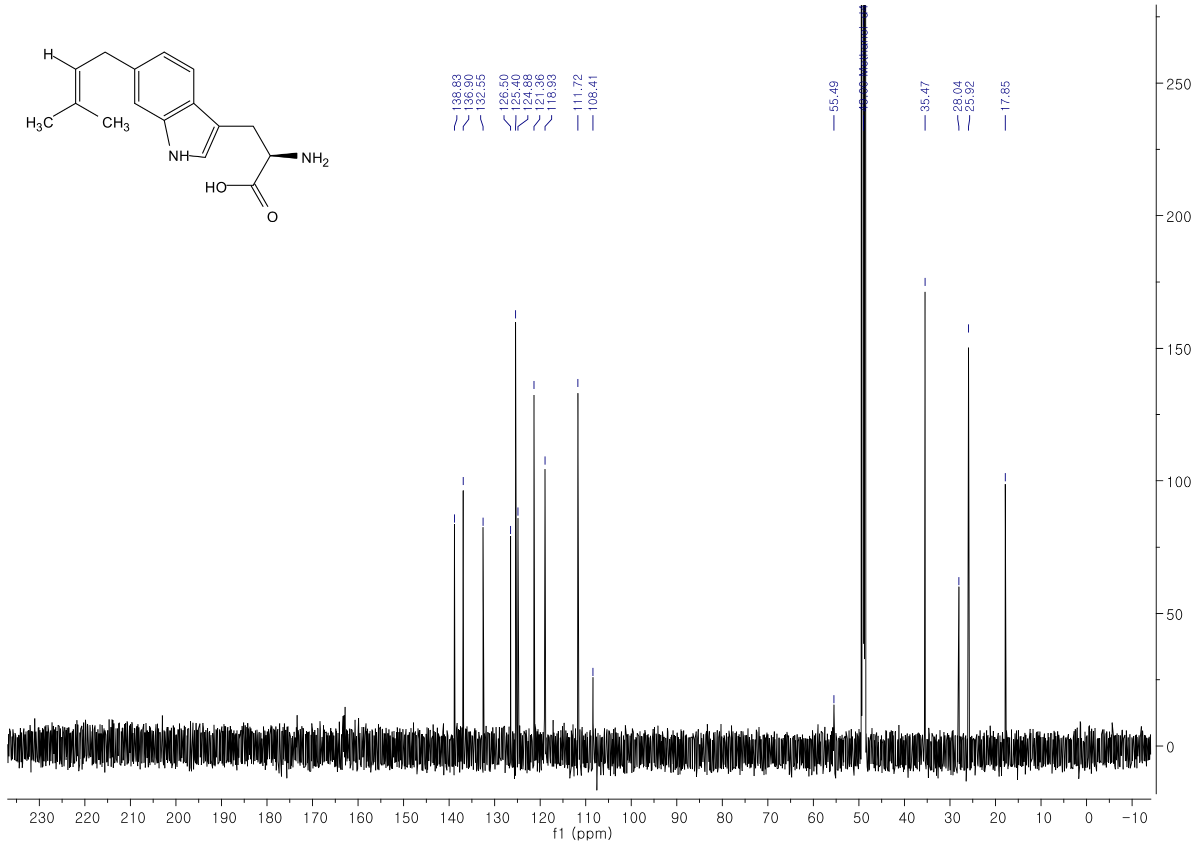

Figure S8. COSY NMR spectrum of 6-dimethylallyl-L-tryptophan (**2**) at 600 MHz in CD_3OD-d_4.

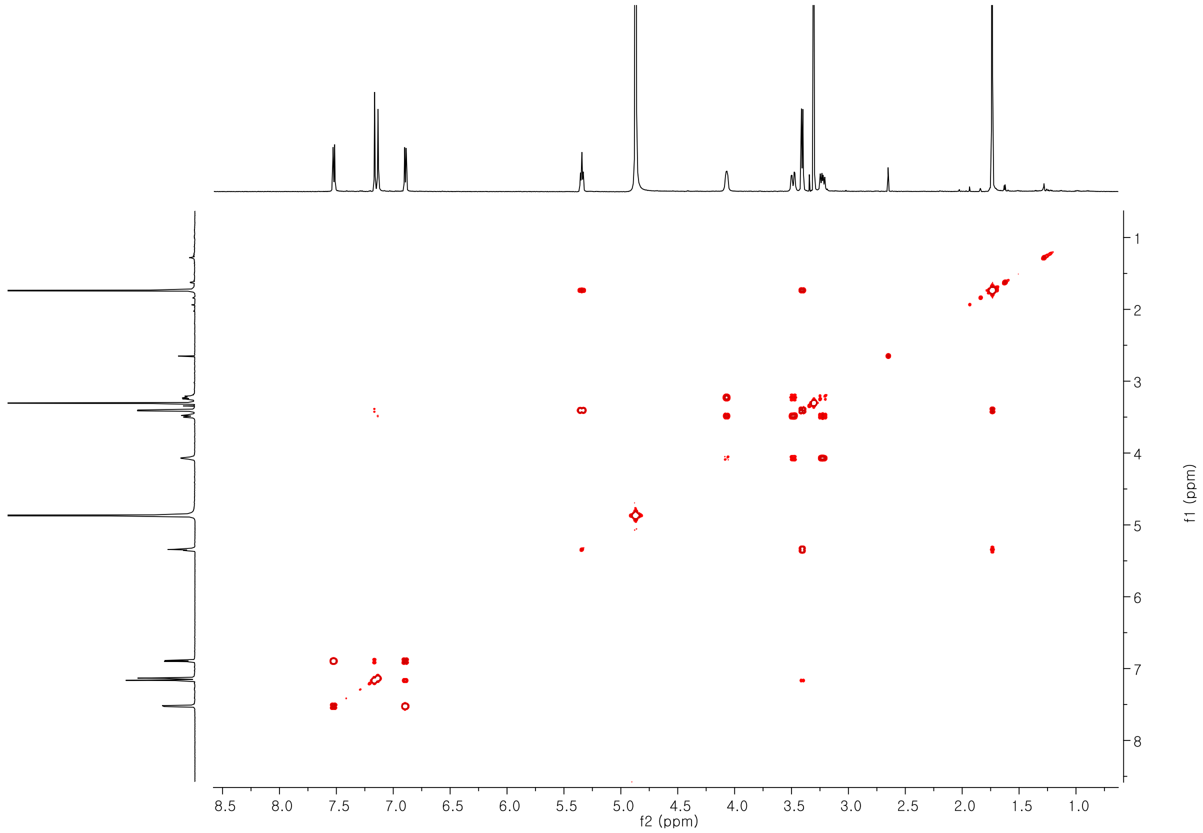

Figure S9. HSQC NMR spectrum of 6-dimethylallyl-L-tryptophan (**2**) at 600 MHz in CD_3OD-d_4.

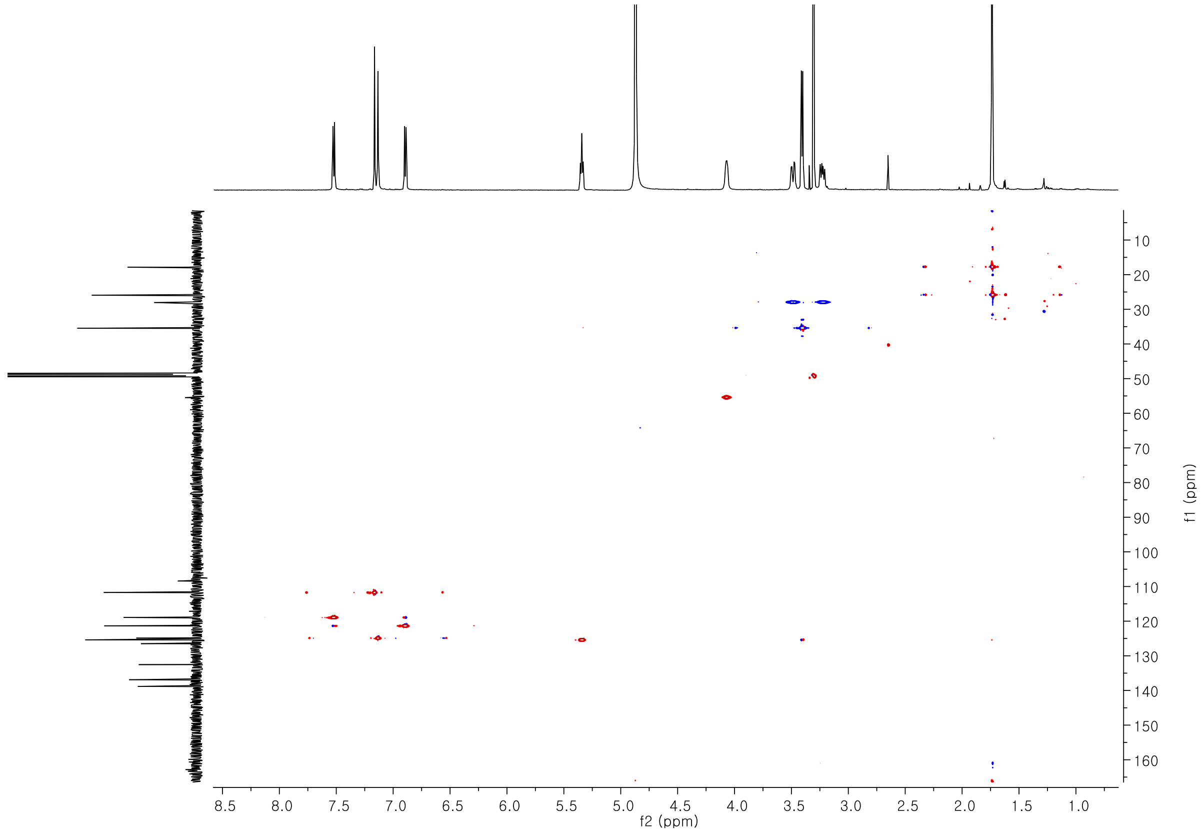

Figure S10. HMBC NMR spectrum of 6-dimethylallyl-L-tryptophan (**2**) at 600 MHz in CD_3OD-d_4.

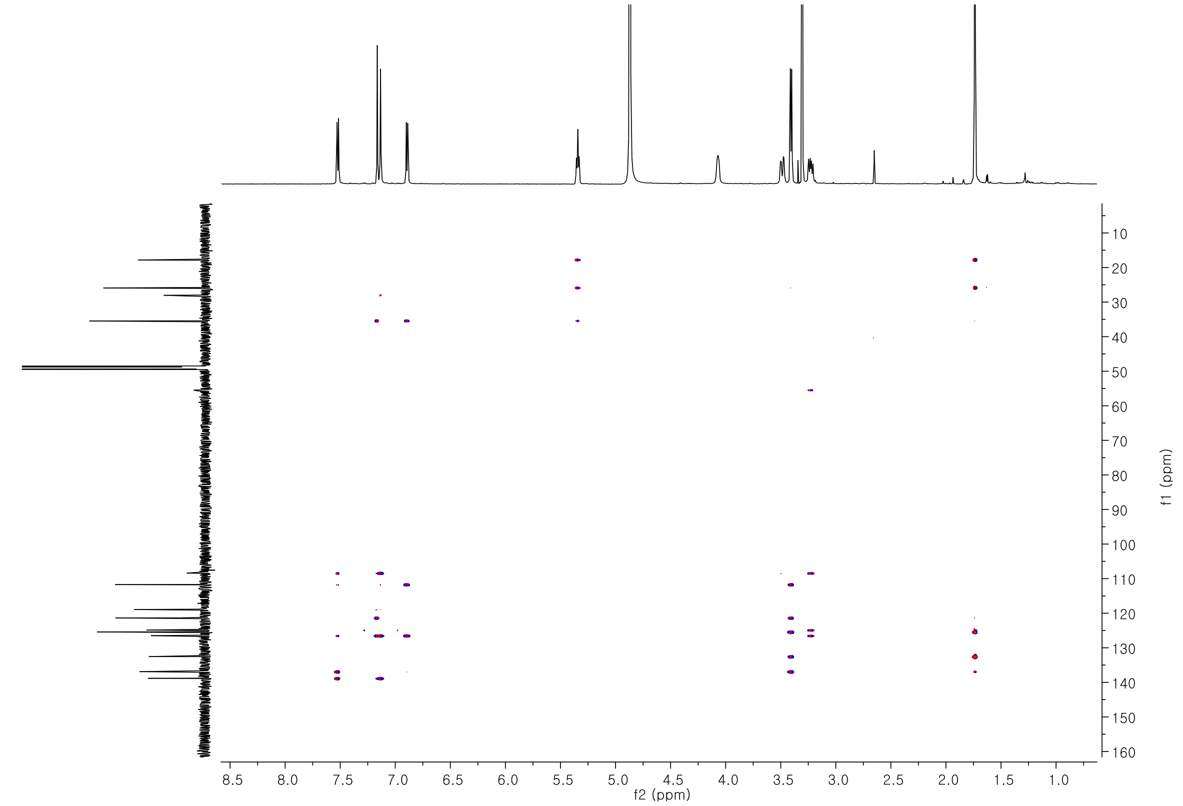

Figure S11. ^{1}H NMR spectrum of penipaline D (**3**) at 700 MHz in CD_3OD-d_4.

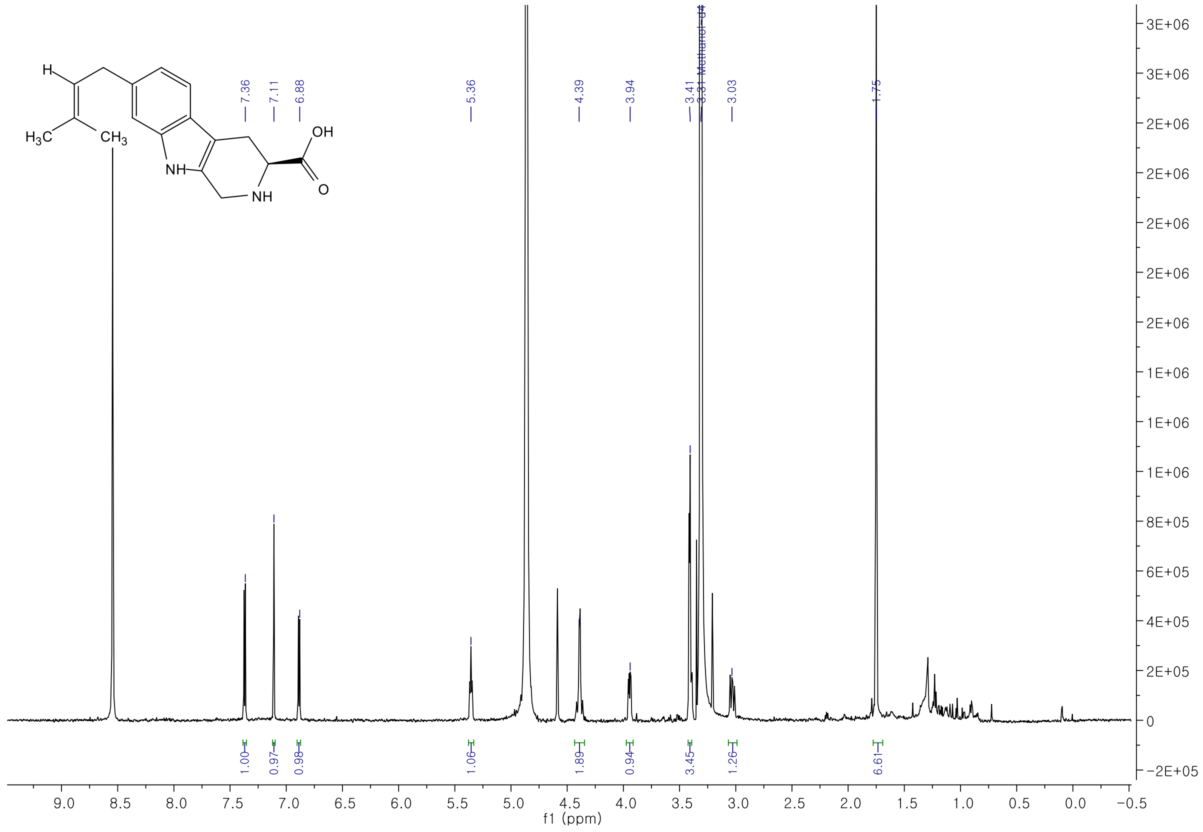

Figure S12. ^{13}C NMR spectrum of penipaline D (**3**) at 175 MHz in CD_3OD-d_4.

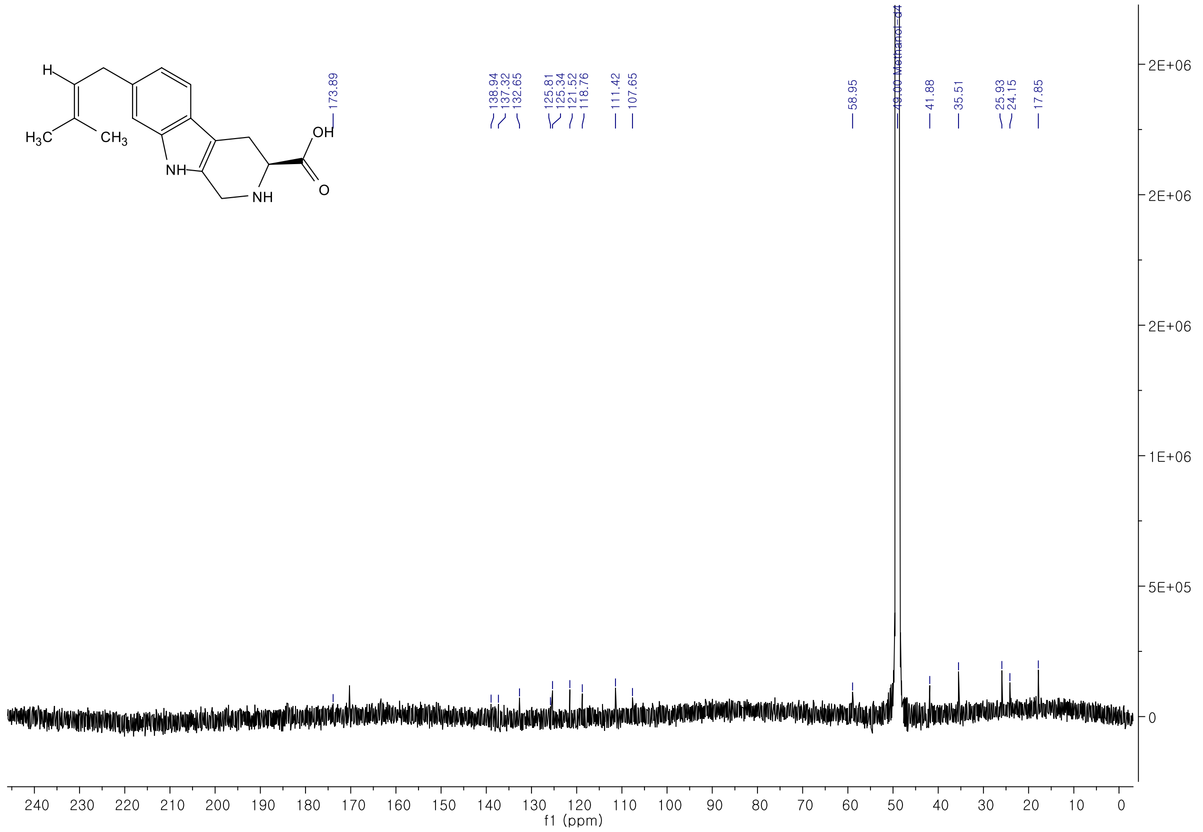

Figure S13. COSY NMR spectrum of penipaline D (**3**) at 700 MHz in CD_3OD-d_4.

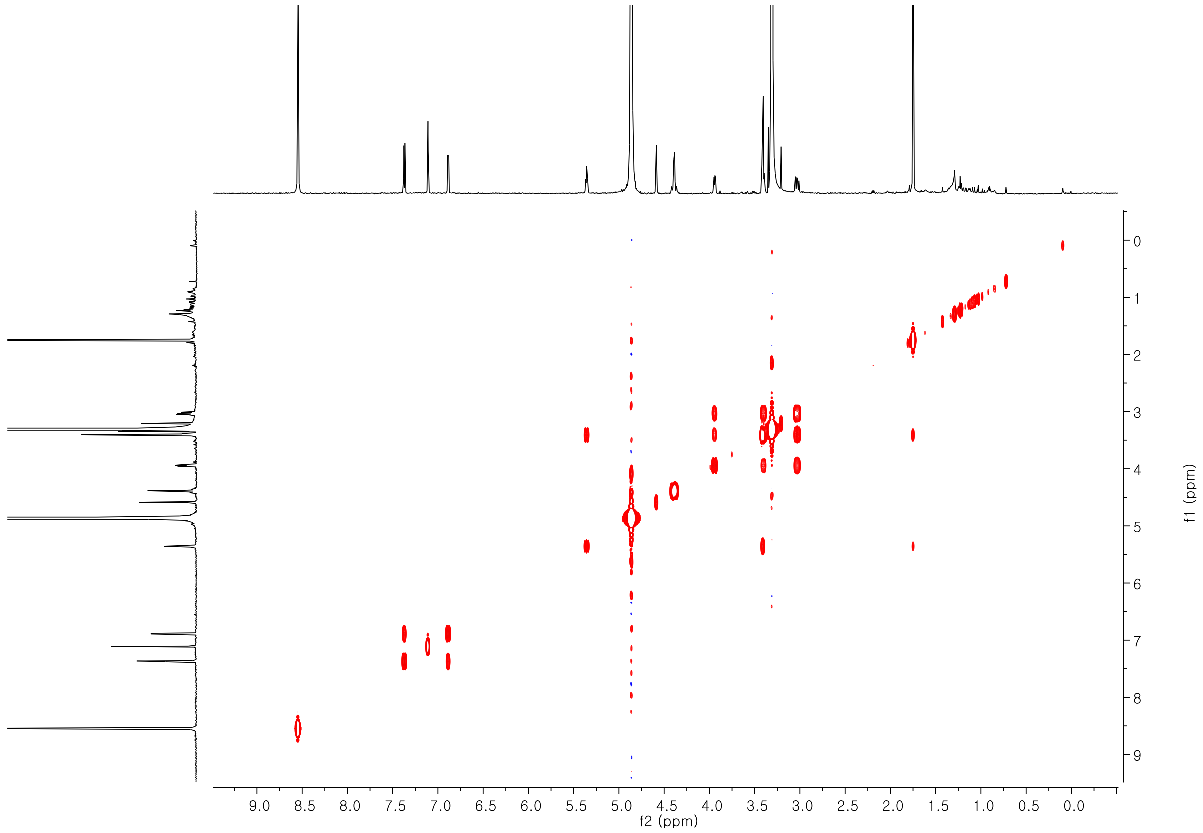

Figure S14. HSQC NMR spectrum of penipaline D (**3**) at 700 MHz in CD_3OD-d_4.

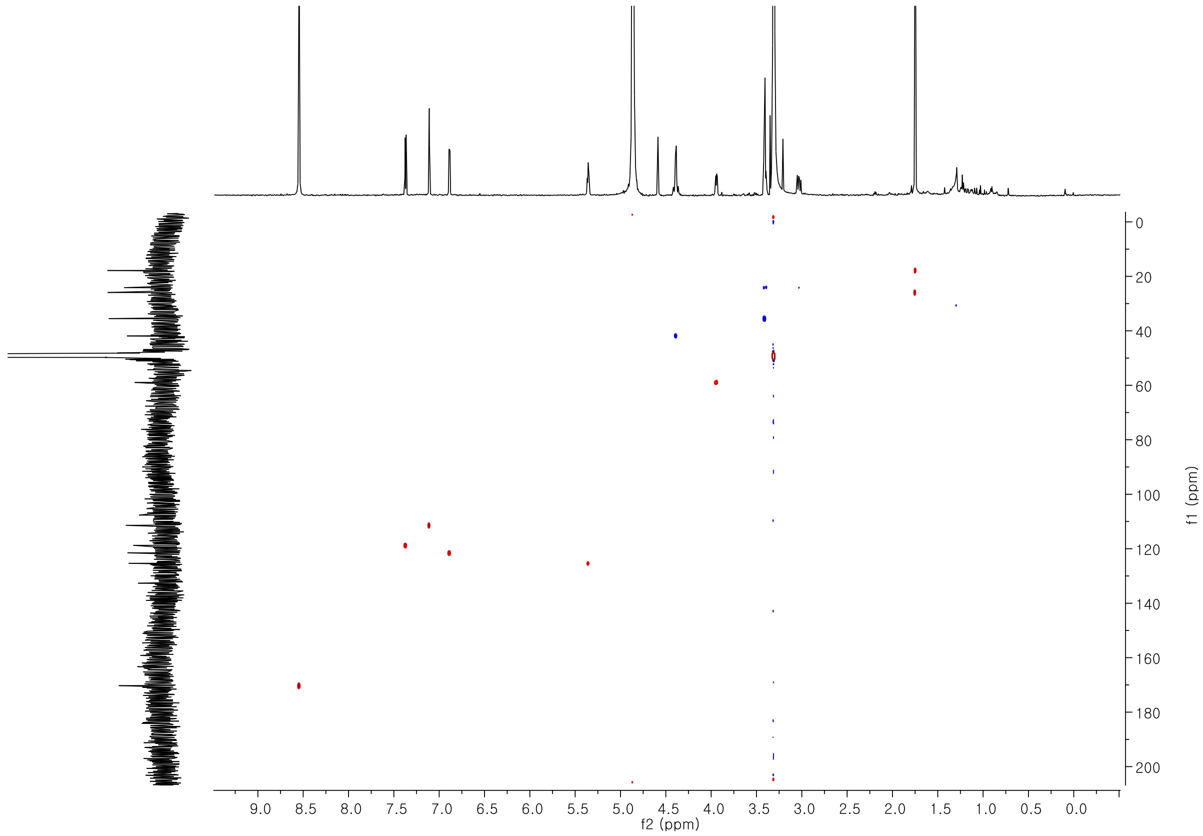

Figure S15. HMBC NMR spectrum of penipaline D (**3**) at 700 MHz in CD_3OD-d_4.

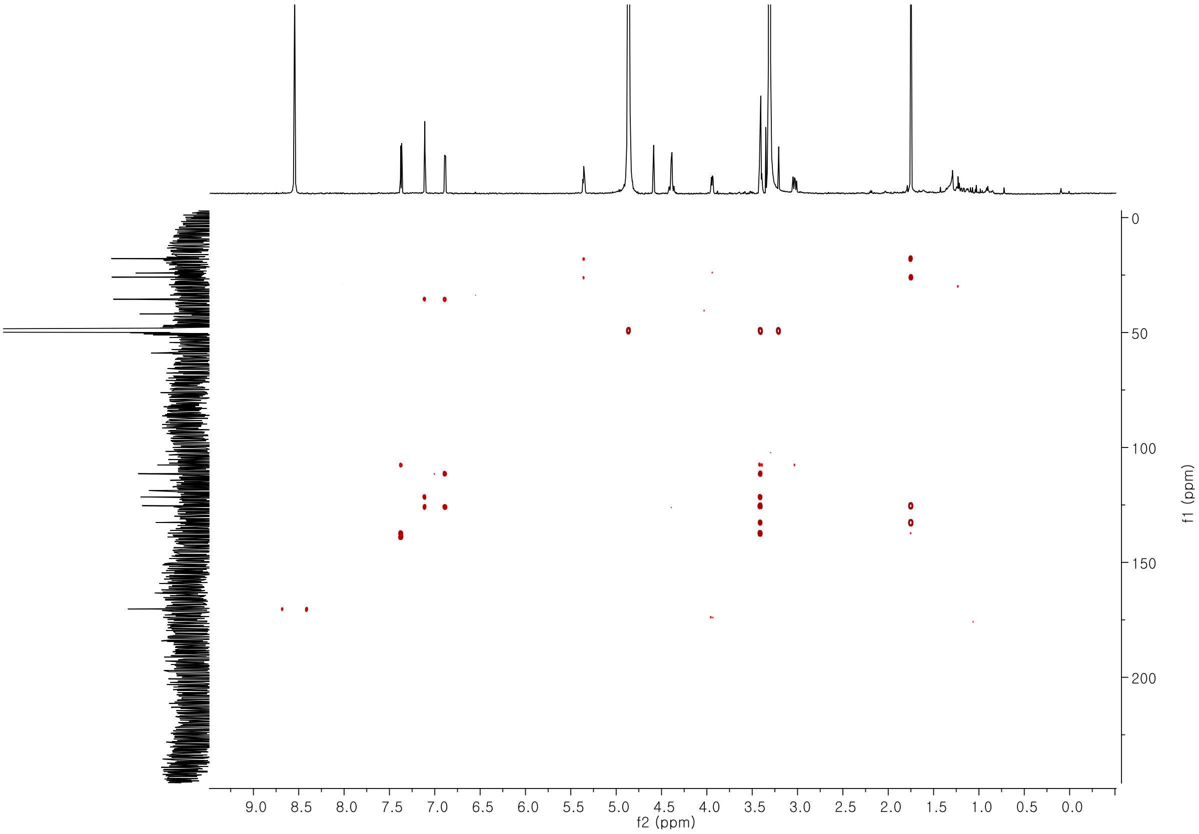

Figure S16. ^{1}H NMR spectrum of *N*-acetyl-6-dimethylallyl-L-tryptophan (**4**) at 900 MHz in CD_3OD-d_4.

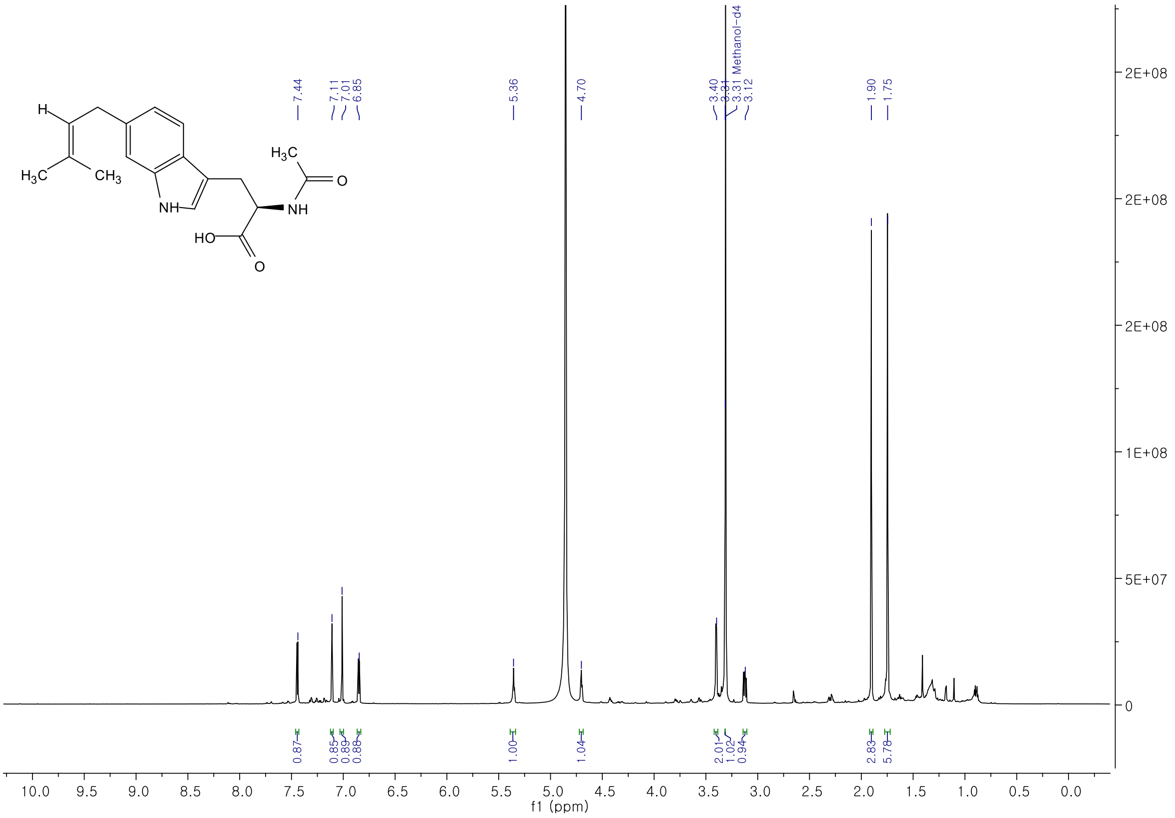

Figure S17. ^{13}C NMR spectrum of *N*-acetyl-6-dimethylallyl-L-tryptophan (**4**) at 225 MHz in CD_3OD-*d*$_4$.

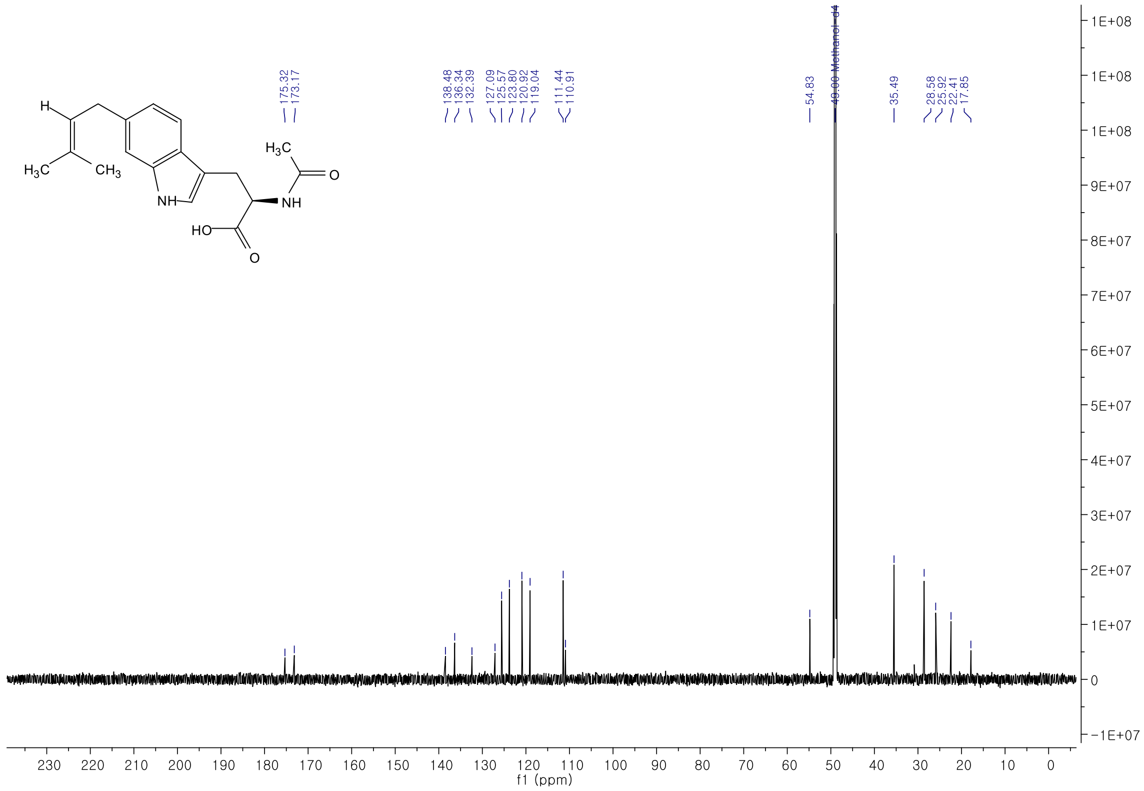

Figure S18. COSY NMR spectrum of *N*-acetyl-6-dimethylallyl-L-tryptophan (**4**) at 900 MHz in CD_3OD-*d*$_4$.

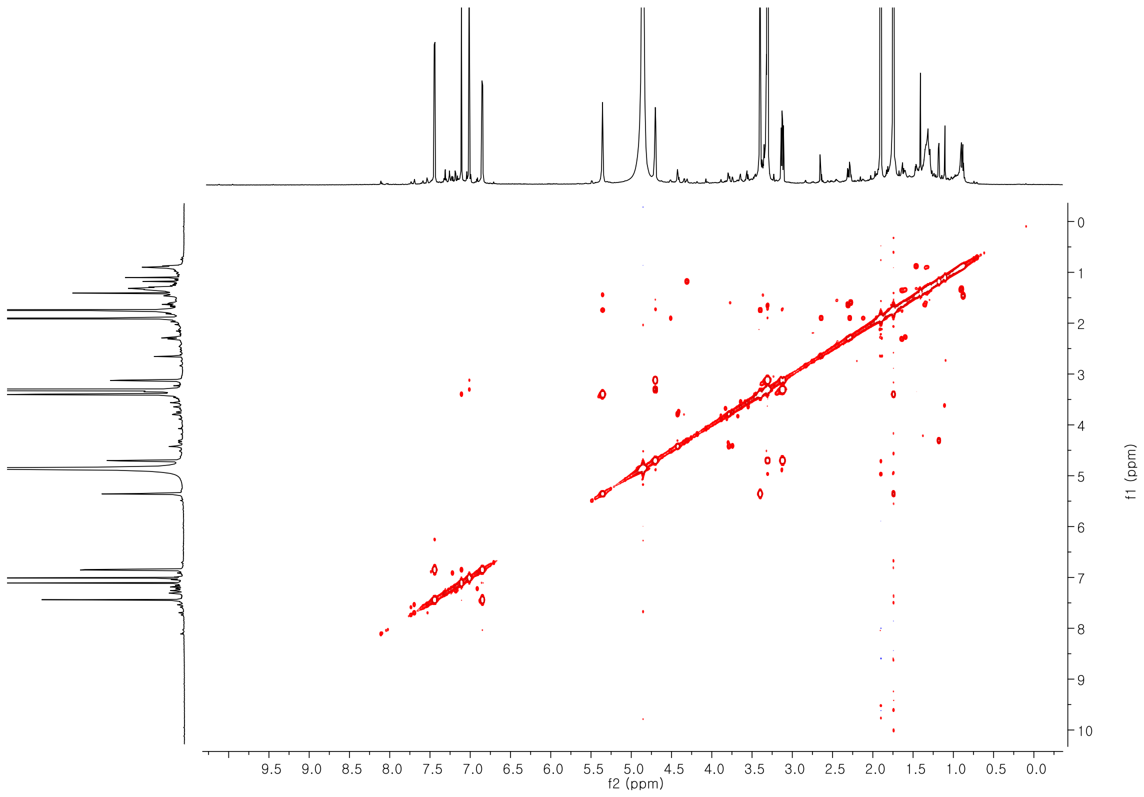

Figure S19. HSQC NMR spectrum of *N*-acetyl-6-dimethylallyl-L-tryptophan (**4**) at 900 MHz in CD_3OD-d_4.

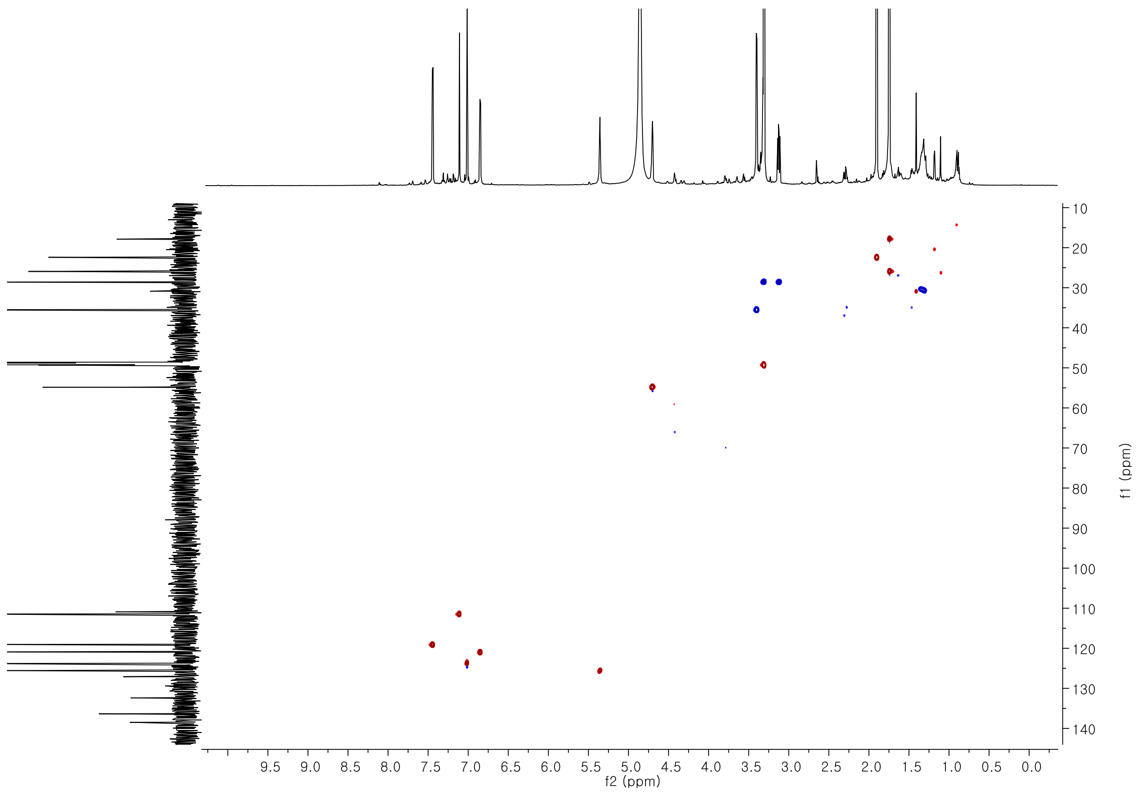

Figure S20. HMBC NMR spectrum of *N*-acetyl-6-dimethylallyl-L-tryptophan (**4**) at 900 MHz in CD_3OD-d_4.

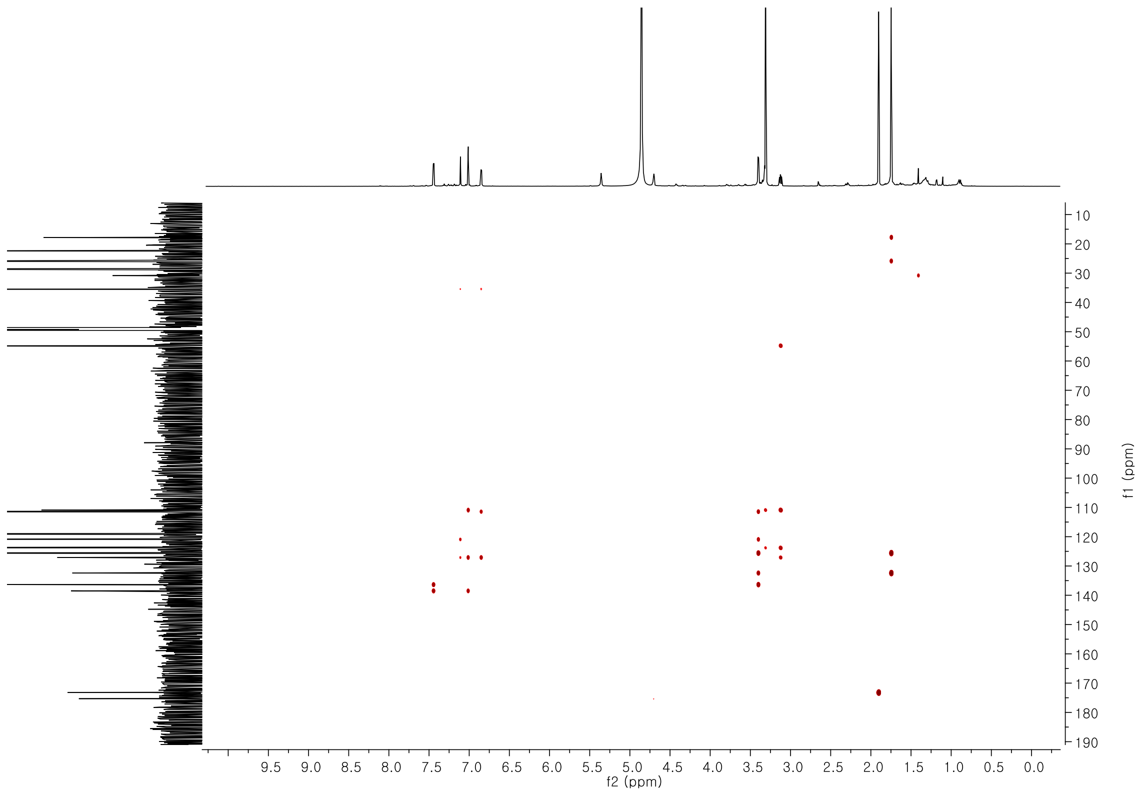

Figure S21. HR-ESI-MS data of 6-dimethylallyl-indole (**1**).

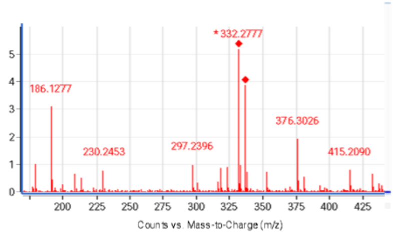

Figure S22. HR-ESI-MS data of 6-dimethylallyl-L-tryptophan (**2**).

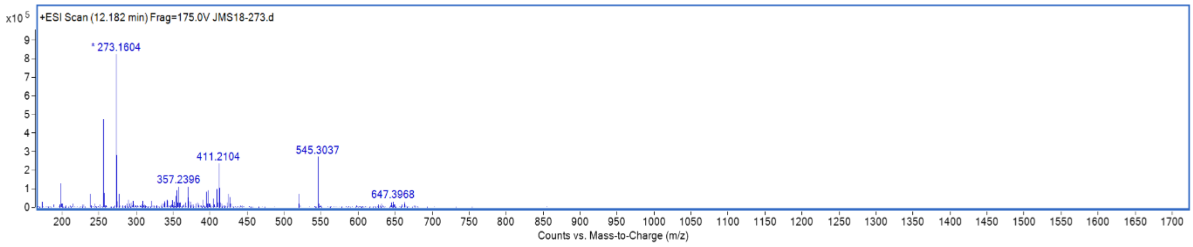

Figure S23. HR-ESI-MS data of penipaline D (**3**).

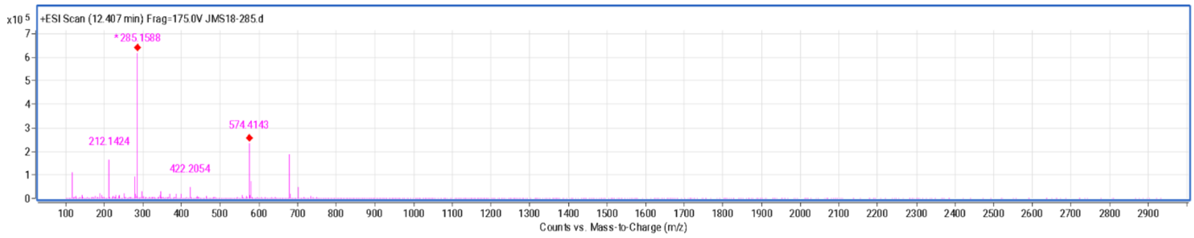

Figure S24. HR-ESI-MS data of *N*-acetyl-6-dimethylallyl-L-tryptophan (**4**).

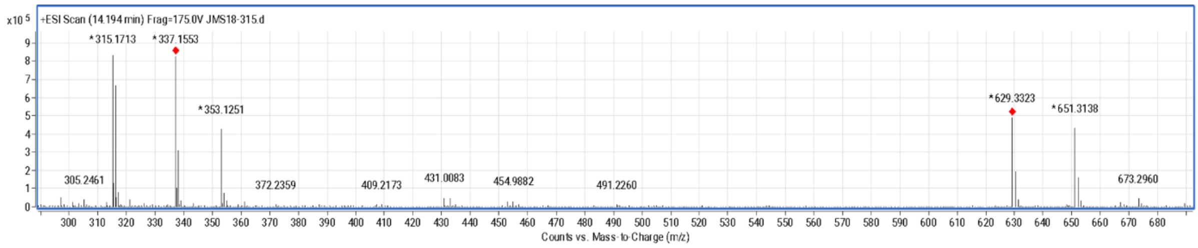

Figure S25. Experimental and calculated ECD spectra of (*R*)- and (*S*)-6-dimethylallyl-L-tryptophan (**2**).

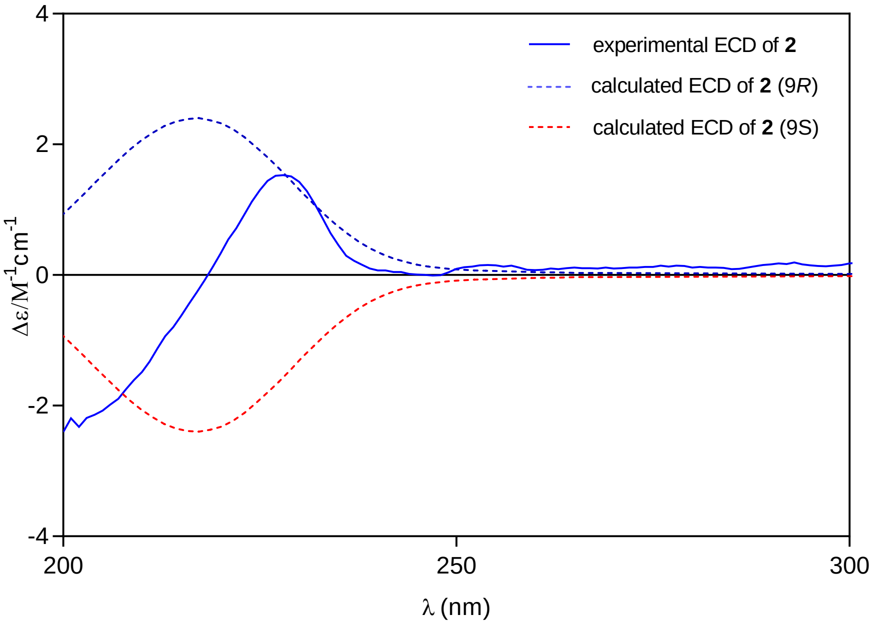

Figure S26. Experimental and calculated ECD spectra of (*R*)- and (*S*)- *N*-Acetyl-6-dimethylallyl-L-tryptophan (**4**).

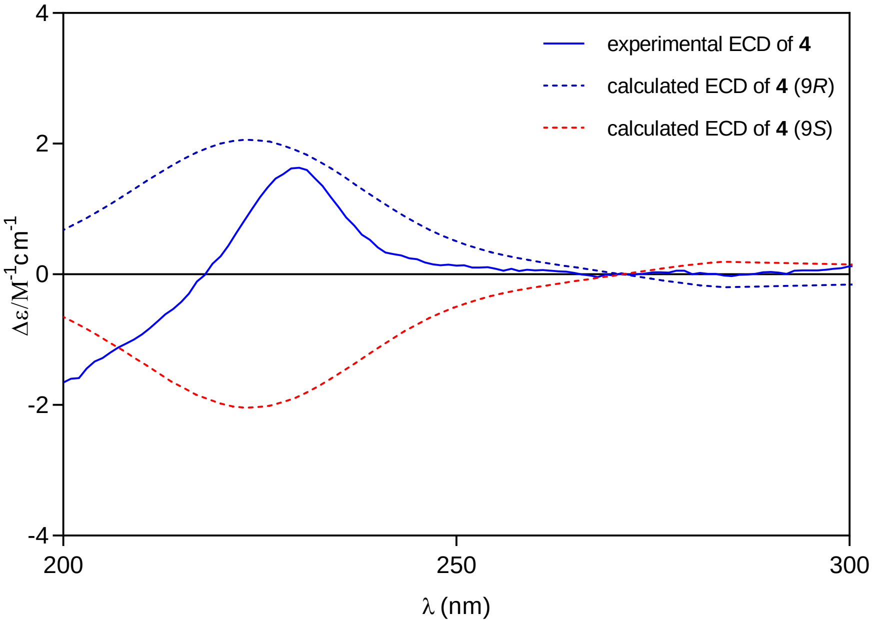

Table S1. ECD calculation of (*R*)- 6-dimethylallyl-L-tryptophan (**2**).

total energy = -881.52072625424

kinetic energy = 877.03702215275

potential energy = -1758.55774840699

Parameters of Level DFT

DFT settings (Functional B3LYP / Gridsize M3)

Geometry optimization options (Energy 10-6 Hartree, Gradient norm │dE / dxyz│= 10-3 Hartree/Bohr)

Energy minimized coordinates of (*R*)- 6-dimethylallyl-L-tryptophan (**2**) at the basis set def-TZVPP) for all atoms (Å).

Atom	X	Y	Z
C	-7.5162	-1.1131	-1.1431
C	-6.551	-0.4218	-0.2097
H	-8.435	-0.5135	-1.2755
H	-7.8397	-2.0863	-0.7316
H	-7.0775	-1.2889	-2.1366
C	-7.1006	-0.1579	1.1706
H	-7.9809	0.5077	1.1171
H	-6.373	0.3031	1.8521
H	-7.4532	-1.0966	1.6341
C	-5.3178	-0.0768	-0.6268
C	-4.2345	0.6362	0.1387
H	-5.0476	-0.3326	-1.6594
C	-2.9208	-0.1296	0.2421
H	-4.5786	0.8931	1.155
H	-4.0266	1.6034	-0.354

C	-2.9129	-1.5141	0.4221
C	-1.6783	-2.1637	0.557
C	-0.4387	-1.4664	0.5053
C	-0.4636	-0.0692	0.3257
C	-1.6893	0.5734	0.1954
H	-3.8527	-2.0711	0.4565
H	0.4651	0.5063	0.2891
H	-1.7107	1.6578	0.0536
C	0.6144	-2.4471	0.6783
N	-1.389	-3.498	0.7563
C	-0.0224	-3.6647	0.8301
H	0.4103	-4.6501	0.9996
H	-2.071	-4.2416	0.8255
C	2.0934	-2.1794	0.7327
C	2.7657	-1.7639	-0.6057
H	2.6189	-3.0608	1.1391
H	2.295	-1.3494	1.4289
C	2.8643	-2.9329	-1.6102
O	1.7166	-3.5812	-1.886
O	3.8949	-3.2468	-2.1467
N	4.0522	-1.1469	-0.3378
H	2.1159	-1.0055	-1.0818
H	1.0017	-3.2662	-1.3011
H	4.6862	-1.859	0.0336
H	4.4796	-0.8779	-1.2255

Table S2. ECD calculation of (*S*)- 6-dimethylallyl-L-tryptophan (**2**).

total energy = -881.52072625396

kinetic energy = 877.03702224767

potential energy = -1758.55774850163

Parameters of Level DFT

DFT settings (Functional B3LYP / Gridsize M3)

Geometry optimization options (Energy 10-6 Hartree, Gradient norm ｜dE / dxyz｜= 10-3 Hartree/Bohr)

Energy minimized coordinates of (*S*)- 6-dimethylallyl-L-tryptophan (**2**) at the basis set def-TZVPP for all atoms (Å).

Atom	X	Y	Z
C	7.5162	-1.1131	-1.1431
C	6.551	-0.4218	-0.2097
H	8.435	-0.5135	-1.2755
H	7.8397	-2.0863	-0.7316
H	7.0775	-1.2889	-2.1366
C	7.1006	-0.1579	1.1706
H	7.9809	0.5077	1.1171
H	6.373	0.3031	1.8521
H	7.4532	-1.0966	1.6341
C	5.3178	-0.0768	-0.6267
C	4.2345	0.6362	0.1387
H	5.0475	-0.3326	-1.6594
C	2.9208	-0.1296	0.2421
H	4.5786	0.8931	1.155
H	4.0266	1.6034	-0.354
C	2.9129	-1.5141	0.4221

C	1.6783	-2.1637	0.5571
C	0.4387	-1.4664	0.5053
C	0.4636	-0.0692	0.3257
C	1.6893	0.5734	0.1954
H	3.8527	-2.0711	0.4565
H	-0.4651	0.5063	0.2891
H	1.7107	1.6578	0.0536
C	-0.6144	-2.4471	0.6783
N	1.389	-3.498	0.7563
C	0.0224	-3.6647	0.8301
H	-0.4103	-4.6501	0.9996
H	2.071	-4.2416	0.8255
C	-2.0934	-2.1794	0.7327
C	-2.7657	-1.7639	-0.6057
H	-2.6189	-3.0608	1.1391
H	-2.295	-1.3494	1.4289
C	-2.8643	-2.9329	-1.6102
O	-1.7166	-3.5812	-1.886
O	-3.8949	-3.2468	-2.1467
N	-4.0522	-1.1469	-0.3378
H	-2.1159	-1.0055	-1.0818
H	-1.0017	-3.2662	-1.3011
H	-4.6862	-1.859	0.0336
H	-4.4796	-0.8779	-1.2255

Table S3. ECD calculation of (*R*)-*N*-Acetyl-6-dimethylallyl-L-tryptophan (**4**).

total energy = -1034.15529952660

kinetic energy = 1028.97179617770

potential energy = -2063.12709570430

Parameters of Level DFT

DFT settings (Functional B3LYP / Gridsize M3)

Geometry optimization options (Energy 10-6 Hartree, Gradient norm ｜dE / dxyz｜= 10-3 Hartree/Bohr)

Energy minimized coordinates of (*R*)-N-Acetyl-6-dimethylallyl-L-tryptophan (**4**) at the basis set def-TZVPP for all atoms (Å).

Atom	X	Y	Z
C	-8.8683	-2.2517	-0.8561
C	-7.8441	-1.3697	-0.1824
H	-9.1653	-3.0895	-0.1991
H	-8.4948	-2.6725	-1.8015
H	-9.7939	-1.6883	-1.0739
C	-8.3045	-0.7656	1.1217
H	-9.1838	-0.1163	0.9597
H	-7.533	-0.1667	1.624
H	-8.6294	-1.5556	1.822
C	-6.6383	-1.1605	-0.7445
C	-5.5067	-0.3036	-0.2431
H	-6.4341	-1.6678	-1.6967
C	-4.1884	-1.0405	-0.036
H	-5.7865	0.2	0.6981
H	-5.3292	0.5109	-0.9689

C	-4.1658	-2.3699	0.3915
C	-2.9626	-0.3561	-0.2403
C	-1.7307	-0.9631	-0.0219
C	-1.6894	-2.3042	0.4084
C	-2.925	-2.9846	0.6076
H	-5.1011	-2.9129	0.5506
H	-2.9933	0.6839	-0.5786
H	-0.8079	-0.3994	-0.1873
C	-0.6317	-3.2375	0.7279
C	-1.2519	-4.4139	1.0907
N	-2.6209	-4.2635	1.0223
H	-3.2957	-4.9849	1.238
H	-0.7994	-5.362	1.3727
C	0.8459	-2.9696	0.6444
N	2.6439	-2.6128	-1.1444
C	1.8062	-4.9099	-0.7571
O	2.5951	-5.2615	-1.7867
O	1.3735	-5.7221	0.0148
C	3.8626	-2.4344	-0.4839
C	4.311	-3.5209	0.4764
H	3.5365	-3.8028	1.2033
H	5.1994	-3.1565	1.0062
H	4.58	-4.4299	-0.0861
O	4.5525	-1.4712	-0.7479
H	2.9012	-4.4414	-2.2168
C	1.4895	-3.4006	-0.698
H	1.3683	-3.4652	1.4766

H	1.0218	-1.8879	0.754
H	0.7272	-3.2543	-1.4831
H	2.4216	-1.7903	-1.7016

Table S4. ECD calculation of (*S*)-*N*-Acetyl-6-dimethylallyl-L-tryptophan (**4**).

total energy = -1034.15528359764

kinetic energy = 1028.97115144054

potential energy = -2063.12643503818

Parameters of Level DFT

DFT settings (Functional B3LYP / Gridsize M3)

Geometry optimization options (Energy 10-6 Hartree, Gradient norm ｜dE / dxyz｜= 10-3 Hartree/Bohr)

Energy minimized coordinates of (*S*)-N-Acetyl-6-dimethylallyl-L-tryptophan (**4**) at the basis set def-TZVPP for all atoms (Å).

Atom	X	Y	Z
C	8.8818	-2.2458	-0.8496
C	7.852	-1.3634	-0.1851
H	9.1811	-3.0772	-0.1855
H	8.5127	-2.6754	-1.7928
H	9.8054	-1.6797	-1.0693
C	8.3079	-0.744	1.1132
H	9.178	-0.0841	0.9438
H	7.5299	-0.1518	1.6133
H	8.6456	-1.5243	1.8182
C	6.6459	-1.1659	-0.751
C	5.509	-0.3089	-0.2619
H	6.4466	-1.6836	-1.6987
C	4.1912	-1.0462	-0.0538
H	5.7835	0.2039	0.6759
H	5.3328	0.4991	-0.9954

C	4.1683	-2.3765	0.3704
C	2.9653	-0.3604	-0.2542
C	1.7334	-0.9668	-0.0343
C	1.6918	-2.3088	0.3935
C	2.9273	-2.9906	0.5883
H	5.1032	-2.9208	0.5264
H	2.9961	0.6801	-0.591
H	0.8107	-0.402	-0.1966
C	0.6341	-3.2416	0.7143
C	1.2543	-4.4193	1.0734
N	2.6232	-4.2699	1.0012
H	3.298	-4.9921	1.2144
H	0.8013	-5.3669	1.3563
C	-0.8436	-2.9714	0.6378
N	-2.6507	-2.6349	-1.1464
C	-1.8311	-4.9313	-0.7168
O	-2.638	-5.2959	-1.7275
O	-1.3929	-5.7321	0.0639
C	-3.8613	-2.4262	-0.4798
C	-4.3105	-3.4807	0.515
H	-3.5363	-3.735	1.2526
H	-5.2	-3.0995	1.031
H	-4.5768	-4.4095	-0.0154
O	-4.5439	-1.4634	-0.7635
H	-2.9424	-4.4813	-2.1695
C	-1.5007	-3.424	-0.6912
H	-1.3604	-3.4508	1.4829

H	-1.0153	-1.8875	0.73
H	-0.7424	-3.3011	-1.4842
H	-2.425	-1.8296	-1.727

Table S5. Cartesian coordinates of (*R*)- 6-dimethylallyl-L-tryptophan (**2**).

No.	Atom	Cartesian coordinates		
1	C	-7.5217	-1.3426	-0.3749
2	C	-6.2919	-0.6911	0.2016
3	H	-8.3343	-1.3365	0.3815
4	H	-7.2901	-2.3936	-0.6476
5	H	-7.8901	-0.8217	-1.2846
6	C	-5.7077	-1.319	1.4454
7	H	-6.4729	-1.3197	2.2495
8	H	-4.8158	-0.7905	1.8338
9	H	-5.4167	-2.3678	1.2269
10	C	-5.7727	0.4096	-0.3947
11	C	-4.5484	1.1598	0.0803
12	H	-6.2694	0.8139	-1.2729
13	C	-3.2311	0.4148	-0.0314
14	H	-4.7197	1.4713	1.1333
15	H	-4.4498	2.0826	-0.5312
16	C	-3.1161	-0.8812	-0.5985
17	C	-1.8541	-1.4688	-0.6524
18	C	-0.7382	-0.8326	-0.1799
19	C	-0.8058	0.441	0.3793
20	C	-2.064	1.0637	0.4488
21	H	-3.9665	-1.4206	-0.9906
22	H	0.0805	0.94	0.7495
23	H	-2.1334	2.0546	0.8806
24	C	0.3333	-1.6817	-0.3819
25	N	-1.5383	-2.6773	-1.147

26	C	-0.2028	-2.8184	-0.9888
27	H	0.339	-3.6962	-1.3044
28	H	-2.1912	-3.3722	-1.5728
29	C	1.7659	-1.3809	-0.012
30	C	2.7826	-2.5034	-0.3108
31	H	1.8081	-1.1364	1.072
32	H	2.0663	-0.4737	-0.58
33	C	2.5102	-3.7497	0.5109
34	O	2.2924	-4.9414	-0.0859
35	O	2.5079	-3.6879	1.7306
36	N	4.1346	-2.0139	-0.0327
37	H	2.728	-2.746	-1.3951
38	H	2.3137	-5.0448	-1.0473
39	H	4.24	-1.7815	0.9824
40	H	4.8329	-2.7522	-0.2817

Table S6. Cartesian coordinates of (*S*)- 6-dimethylallyl-L-tryptophan (**2**).

No.	Atom	Cartesian coordinates		
1	C	7.5217	-1.3426	-0.3749
2	C	6.2919	-0.6911	0.2016
3	H	8.3343	-1.3365	0.3815
4	H	7.2901	-2.3936	-0.6476
5	H	7.8901	-0.8217	-1.2846
6	C	5.7077	-1.319	1.4454
7	H	6.4729	-1.3197	2.2495
8	H	4.8158	-0.7905	1.8338
9	H	5.4167	-2.3678	1.2269
10	C	5.7727	0.4096	-0.3947
11	C	4.5484	1.1598	0.0803
12	H	6.2694	0.8139	-1.2729
13	C	3.2311	0.4148	-0.0314
14	H	4.7197	1.4713	1.1333
15	H	4.4498	2.0826	-0.5312
16	C	3.1161	-0.8812	-0.5985
17	C	1.8541	-1.4688	-0.6524
18	C	0.7382	-0.8326	-0.1799
19	C	0.8058	0.441	0.3793
20	C	2.064	1.0637	0.4488
21	H	3.9665	-1.4206	-0.9906
22	H	-0.0805	0.94	0.7495
23	H	2.1334	2.0546	0.8806
24	C	-0.3333	-1.6817	-0.3819
25	N	1.5383	-2.6773	-1.147

26	C	0.2028	-2.8184	-0.9888
27	H	-0.339	-3.6962	-1.3044
28	H	2.1912	-3.3722	-1.5728
29	C	-1.7659	-1.3809	-0.012
30	C	-2.7826	-2.5034	-0.3108
31	H	-1.8081	-1.1364	1.072
32	H	-2.0663	-0.4737	-0.58
33	C	-2.5102	-3.7497	0.5109
34	O	-2.2924	-4.9414	-0.0859
35	O	-2.5079	-3.6879	1.7306
36	N	-4.1346	-2.0139	-0.0327
37	H	-2.728	-2.746	-1.3951
38	H	-2.3137	-5.0448	-1.0473
39	H	-4.24	-1.7815	0.9824
40	H	-4.8329	-2.7522	-0.2817

Table S7. Cartesian coordinates of (*R*)-*N*-Acetyl-6-dimethylallyl-L-tryptophan (**4**).

No.	Atom	Cartesian coordinates		
1	C	-9.2421	-1.2857	0.1025
2	C	-7.7551	-1.5698	0.0529
3	H	-9.858	-2.2032	0.0006
4	H	-9.5209	-0.5976	-0.724
5	H	-9.5016	-0.81	1.0723
6	C	-7.3407	-2.9958	-0.1198
7	H	-8.214	-3.677	-0.1882
8	H	-6.7675	-3.3211	0.7721
9	H	-6.7936	-3.1036	-1.0782
10	C	-6.9233	-0.5006	0.1707
11	C	-5.4152	-0.222	0.1895
12	H	-7.4492	0.4467	0.2884
13	C	-4.1533	-1.0828	0.0747
14	H	-5.2816	0.3127	1.156
15	H	-5.297	0.5275	-0.6243
16	C	-4.0648	-2.4853	-0.1001
17	C	-2.9323	-0.3478	0.1577
18	C	-1.6783	-0.974	0.0724
19	C	-1.6528	-2.3514	-0.0981
20	C	-2.8052	-3.0789	-0.1814
21	H	-4.8905	-3.1333	-0.1765
22	H	-2.954	0.7276	0.291
23	H	-0.7642	-0.3979	0.1382
24	C	-0.5961	-3.235	-0.2128
25	C	-1.182	-4.4915	-0.3709

26	N	-2.5296	-4.3837	-0.3477
27	H	-3.2172	-5.1637	-0.4446
28	H	-0.6701	-5.4305	-0.503
29	C	0.8647	-2.8481	-0.1799
30	N	3.2937	-3.6574	-0.4082
31	C	1.744	-4.946	0.9005
32	O	1.4333	-6.2501	0.7377
33	O	1.9601	-4.5238	2.026
34	C	3.9521	-2.4554	-0.3181
35	C	3.3205	-1.1201	-0.027
36	H	2.6209	-0.8165	-0.8268
37	H	4.102	-0.3304	0.0095
38	H	2.8362	-1.1259	0.9687
39	O	5.1662	-2.4581	-0.4659
40	H	1.2999	-6.641	-0.1369
41	C	1.8738	-4.0161	-0.2879
42	H	1.0291	-2.3028	0.7692
43	H	0.9987	-2.1797	-1.0514
44	H	1.6132	-4.5635	-1.2207
45	H	3.9022	-4.4744	-0.5828

Table S8. Cartesian coordinates of (*S*)-*N*-Acetyl-6-dimethylallyl-L-tryptophan (**4**).

No.	Atom	Cartesian coordinates		
1	C	9.2421	-1.2856	0.102
2	C	7.7551	-1.5698	0.0527
3	H	9.8581	-2.2031	-0.0003
4	H	9.5207	-0.5973	-0.7243
5	H	9.5018	-0.8102	1.072
6	C	7.3407	-2.9957	-0.1204
7	H	8.214	-3.6769	-0.1894
8	H	6.7679	-3.3214	0.7716
9	H	6.7932	-3.1032	-1.0786
10	C	6.9233	-0.5007	0.171
11	C	5.4152	-0.2221	0.19
12	H	7.4492	0.4467	0.2889
13	C	4.1533	-1.0829	0.0752
14	H	5.2817	0.3124	1.1566
15	H	5.297	0.5275	-0.6236
16	C	4.0648	-2.4854	-0.0996
17	C	2.9323	-0.3478	0.1581
18	C	1.6783	-0.974	0.0727
19	C	1.6528	-2.3515	-0.0978
20	C	2.8052	-3.0789	-0.181
21	H	4.8905	-3.1334	-0.1759
22	H	2.954	0.7275	0.2914
23	H	0.7642	-0.398	0.1384
24	C	0.5961	-3.235	-0.2125
25	C	1.182	-4.4916	-0.3705

26	N	2.5296	-4.3837	-0.3473
27	H	3.2172	-5.1637	-0.4441
28	H	0.6701	-5.4305	-0.5027
29	C	-0.8647	-2.8481	-0.1799
30	N	-3.2937	-3.6574	-0.4084
31	C	-1.7442	-4.9459	0.9006
32	O	-1.4334	-6.25	0.7379
33	O	-1.9604	-4.5236	2.026
34	C	-3.9521	-2.4554	-0.3185
35	C	-3.3205	-1.1201	-0.0276
36	H	-2.6208	-0.8166	-0.8274
37	H	-4.1021	-0.3303	0.0086
38	H	-2.8363	-1.1256	0.9681
39	O	-5.1662	-2.4581	-0.4665
40	H	-1.2999	-6.641	-0.1365
41	C	-1.8738	-4.0161	-0.2879
42	H	-1.0292	-2.3027	0.7692
43	H	-0.9986	-2.1798	-1.0514
44	H	-1.6131	-4.5636	-1.2206
45	H	-3.9022	-4.4744	-0.583
